# Bioactive α-Pyrone Analogs from the Endophytic Fungus *Diaporthe* sp. CB10100: α-Glucosidase Inhibitory Activity, Molecular Docking, and Molecular Dynamics Studies

**DOI:** 10.3390/molecules29081768

**Published:** 2024-04-12

**Authors:** Zhong Wang, Qingxian Ma, Guangling Wu, Yani Zhong, Bin Feng, Pingzhi Huang, Aijie Li, Genyun Tang, Xueshuang Huang, Hong Pu

**Affiliations:** 1 Hunan Provincial Key Laboratory for Synthetic Biology of Traditional Chinese Medicine, School of Pharmaceutical Sciences, Hunan University of Medicine, Huaihua 418000, China; 13773980672@163.com (Z.W.); 17608470326@163.com (Q.M.); guangling2024@126.com (G.W.); 18874880070@163.com (Y.Z.); 19918037381@163.com (P.H.); 15274565209@163.com (A.L.); tanggenyun@foxmail.com (G.T.); 2 Huaihua Hospital of Traditional Chinese Medicine, Huaihua 418000, China; txz-1221@163.com

**Keywords:** endophytic fungus, *Diaporthe* sp., α-pyrone, α-glucosidase inhibitor

## Abstract

Two α-pyrone analogs were isolated from the endophytic fungus *Diaporthe* sp. CB10100, which is derived from the medicinal plant *Sinomenium acutum*. These analogs included a new compound, diaporpyrone F (**3**), and a known compound, diaporpyrone D (**4**). The structure of **3** was identified by a comprehensive examination of HRESIMS, 1D and 2D NMR spectroscopic data. Bioinformatics analysis revealed that biosynthetic gene clusters for α-pyrone analogs are common in fungi of *Diaporthe* species. The in vitro α-glucosidase inhibitory activity and antibacterial assay of **4** revealed that it has a 46.40% inhibitory effect on α-glucosidase at 800 μM, while no antibacterial activity against methicillin-resistant *Staphylococcus aureus* (MRSA), *Mycolicibacterium* (*Mycobacterium*) *smegmatis* or *Klebsiella pneumoniae* at 64 μg/mL. Molecular docking and molecular dynamics simulations of **4** with α-glucosidase further suggested that the compounds are potential α-glucosidase inhibitors. Therefore, α-pyrone analogs can be used as lead compounds for α-glucosidase inhibitors in more in-depth studies.

## 1. Introduction

The diabetes mellitus epidemic and its complications greatly threaten global health [1,2]. More than 90% of diabetes patients have type 2 diabetes mellitus (T2DM) [3]. Currently, α-glucosidase inhibitors are the primary therapeutic approaches for type 2 diabetes mellitus [4]. In conventional medicine, the only FDA-approved α-glucosidase inhibitors that are crucial for treating type 2 diabetes are acarbose, miglitol and voglibose [5]. Thus, developing novel α-glucosidase inhibitors is essential.

Endophytes, particularly fungal endophytes, contain a wide range of physiologically active natural substances, including important pharmaceutical compounds [6,7,8,9]. Various species of *Diaporthe* fungi, specifically the anamorph *Phomopsis*, have been found as nonpathogenic endophytes, plant pathogens, or parasites on plants worldwide [10]. These fungi are recognized for their remarkable ability to produce bioactive metabolites, such as pyrones, polyketides, and terpenoids [10]. A significant number of these chemicals exhibit antidiabetic, antibacterial, cytotoxic, and anti-inflammatory properties [10]. For example, twelve novel austalide meroterpenoids, named diaporaustalides A−L, were extracted from *Diaporthe* sp. XC1211. Among them, diaporaustalide B and E exhibited strong inhibitory effects on the proliferation of B cells caused by LPS, with IC_50_ values of 6.7 and 3.8 μM, respectively [11]. Phomopthane A was extracted from *Diaporthe unshiuensis* YSP3 and showed cytotoxic effects on HeLa and MCF-7 cells, with IC_50_ values of 5.92 and 7.50 μM, respectively [12]. Cytosporone B obtained from *Diaporthe* sp. IQ-053 had minimum inhibitory concentration (MIC) values of 21 ± 2.6 and 18 ± 2.9 μg/mL against *S. epidermidis* 42R and *S. aureus*, respectively [13].

**Figure 1:**
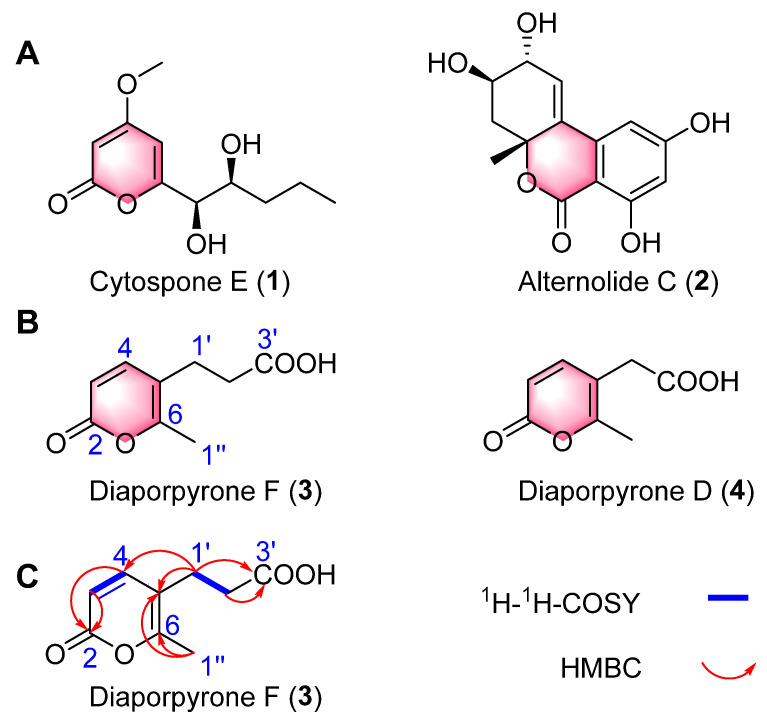
(**A**) Cytospone E (**1**) isolated from the endophytic fungus *Cytospora rhizophorae* A761 and alternolide C (**2**) isolated from the marine-derived fungus *Alternaria alternata* LW37. (**B**) Structures of compounds **3***–***4** isolated from the endophilic fungus *Diaporthe* sp. CB10100. (**C**) Key ^1^H-^1^H COSY and HMBC correlations of diaporpyrone F (**3**).

**Figure 2:**
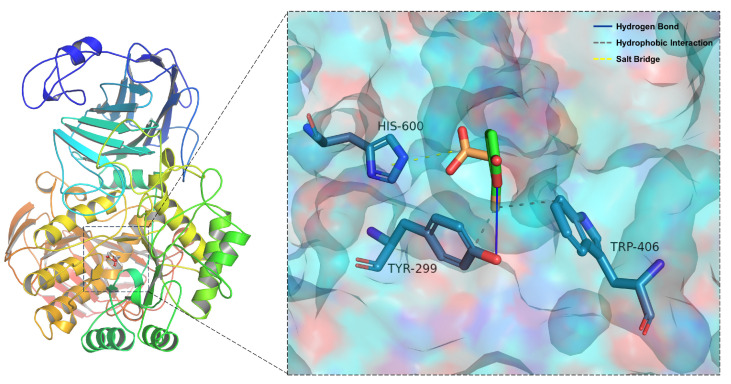
Docking poses and interactions of **4** with α-glucosidase (PDB ID: 2QMJ).

α-Pyrone, an aromatic unsaturated lactone, is present as a substructure in a diverse range of natural substances that exhibit noteworthy biological activities [14]. Many α-pyrones with anti-diabetes effects have been discovered from *Diaporthe* species [10,15,16]. For example, cytospone E (Figure 1A, **1**) derived from the endophytic fungus *Cytospora rhizophorae* A761 showed a 41.0% rate of α-glucosidase inhibition at 100 μM; in contrast, acarbose inhibited α-glucosidase by 34.5% at the same concentration [15]. Alternolide C (Figure 1A, **2**), isolated from the marine-derived fungus *Alternaria alternata* LW37, was demonstrated to exhibit inhibitory effects against α-glucosidase, with an IC_50_ value of 451.25 ± 6.95 μM [16]. As part of our ongoing inquiry into the secondary metabolites produced by endophytic fungi, we discovered a number of intriguing natural products in *Diaporthe* sp. CB10100 that was isolated from *Sinomenium acutum*. These products consist of ellagic acid B, a dibenzo-α-pyrone derivative, and four α-pyrones, diaporpyrones A–D (**S1**–**S4**) [17].

**Figure 3:**
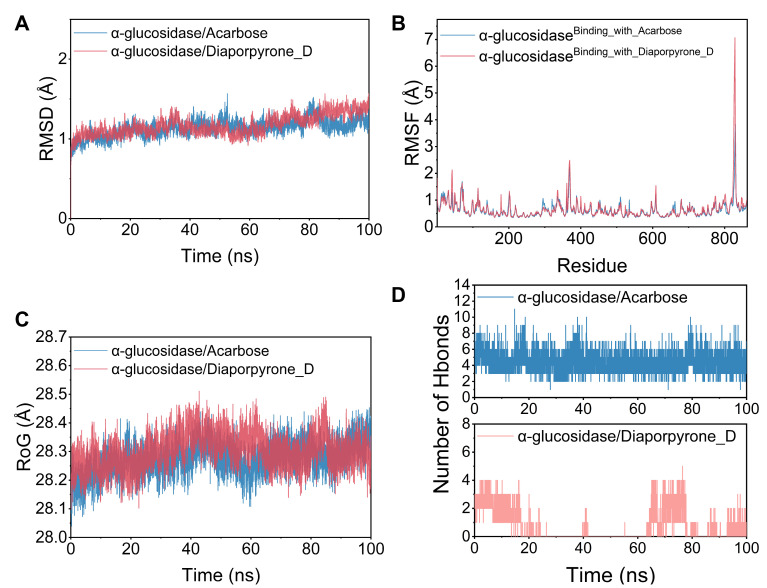
Molecular dynamics of acarbose and **4** with residues in the active pocket of α-glucosidase. (**A**) RMSD values of the complex and protein backbone systems in the dynamic simulation. (**B**) RMSF change profiles of binding site residues in the free protein and complex systems. (**C**) The gyration radius of the four systems during the molecular dynamics simulation. (**D**) The number of hydrogen bonds in the molecular dynamics simulation.

In the present work, further chemical studies were performed with this fungus to identify novel bioactive chemicals. In this study, an undescribed α-pyrone derivative named diaporpyrone F (Figure 1B, **3**) was isolated from the endophytic *Diaporthe* sp. CB10100, as well as a known compound, diaporpyrone D (Figure 1B, **4**). The structures of the new natural compounds were established with full confidence using NMR and HRESIMS. An in vitro α-glucosidase inhibitory activity and antibacterial assay revealed that **4** exhibits a 46.40% inhibitory effect on α-glucosidase at 800 μM but shows no antibacterial activity against MRSA, *Mycolicibacterium* (*Mycobacterium*) *smegmatis* or *Klebsiella pneumoniae* at 64 μg/mL. Molecular docking and molecular dynamics simulations of **4** with α-glucosidase further suggested that **4** is a potential α-glucosidase inhibitor. Our investigation indicated that compound **4**, together with the commonly used α-pyrone scaffold, shows potential as a powerful inhibitor of α-glucosidase.

## 2. Results and Discussion

### 2.1. Structural Elucidation

Crude extracts of *Diaporthe* sp. CB10100 were fractionated using silico gel and MCI highly porous polymers as well as semipreparative HPLC to yield compounds **3**–**4**. Diaporpyrone F (**3**) was isolated as a colorless gum. Its molecular formula was established as C_9_H_10_O_4_ based on (+)-HRESIMS analysis (Figure S9) at *m*/*z* 183.06487 [M + H]^+^ (calcd for C_9_H_11_O_4_, 183.06519), suggesting five degrees of unsaturation. The ^13^C NMR spectrum of **3** (Table 1), DEPT-135 and DEPT-90 show a total of nine signals corresponding to a carboxyl (*δ*_C_ 174.6), an ester carbonyl (*δ*_C_ 161.8), two nonprotonated carbons (*δ*_C_ 158.9, 114.7), two olefinic methine carbons (*δ*_C_ 148.1, 112.4), two methylene carbons (*δ*_C_ 34.8, 24.7), and one methyl carbon (*δ*_C_ 17.1) (Figures S1–S8). The ^1^H NMR spectrum (Table 1) also indicated the presence of one methyl group [*δ*_H_ 2.20 (3H, s)], two methylenes [*δ*_H_ 2.33 (2H, t, *J* = 7.4 Hz and 2.49 (m)] and two sp^2^ methines [*δ*_H_ 6.12 (1H, d, *J* = 9.3 Hz, H-3) and 7.46 (1H, d, *J* = 9.4 Hz, H-4)]. The ^1^H and ^13^C NMR data of **3** are similar to those of a previously isolated pyrone, diaporpyrone D (**4**), with a two-carbon aliphatic chain; one difference was the chemical shift of one carbon atom, C2′ (*δ*_C_ 34.8), indicating the presence of an α-pyrone ring. Based on these observations, relative to diaporpyrone D (**4**), **3** contains one more methylene group (Table 1; Figure S2). This was confirmed by the HMBC (Figure 1C and Figure S6) correlations from H-1″ to C-5 (*δ*_C_ 114.7) and C-6 (*δ*_C_ 158.9), from H-1′ to C-4 (*δ*_C_ 148.1), C-5 (*δ*_C_ 114.7), C-3′ (*δ*_C_ 174.6) and from H-2′ to C-3′ (*δ*_C_ 174.6). The COSY spectrum showed the presence of a spin–spin coupling system consisting of H-1′/H-2′. As a result, the planar structure of **3** was elucidated, and compound **3** was named diaporpyrone F (Figure 1B).

Diaporpyrone D (Figure 1B, **4**) is a known molecule, and its structure was determined by comparing its 1D NMR, 2D NMR, HRESIMS, and UV spectra (Figures S11–S18) with those in the literature [17].

### 2.2. Analysis of Secondary Metabolite Biosynthetic Potential

Five α-pyrone derivatives, including diaporpyrone F (**3**), have been isolated from *Diaporthe* sp. CB10100; thus, we were curious whether similar backbone compounds could be found in other fungi of the genus *Diaporthe* [17]. Whole-genome sequencing has been performed for 18 strains of the genus *Diaporthe* according to the NCBI database [10]. According to the antiSMASH 7.1.0 database, all eighteen of these whole-genome sequences included pyrone biosynthetic gene clusters (Table 2). Surprisingly, the full gene sequence of *Diaporthe* sp. HANT25 contained four clusters of genes for the biosynthesis of α-pyrone analogs. This information will set the stage for further studies on the biosynthesis and synthetic biology of α-pyrone analogs.

### 2.3. α-Glucosidase Inhibition Activity

Since the α-pyrone derivatives cytospone E (**1**) and alternolide C (**2**) have been reported to exhibit α-glucosidase inhibitory activity, we hypothesized that compounds **3**–**4** have α-glucosidase inhibitory activity [15,16]. We evaluated the inhibitory activity of **3** and **4** against α-glucosidase at 800 μM. The assay results showed that **4** inhibited α-glucosidase by 46.4% at a concentration of 800 μM, while acarbose inhibited 57.33% at the same concentration (Table 3). Thus, the compound **4** show potential as lead compounds for the discovery of α-glucosidase inhibitors.

### 2.4. Antibacterial Assay

Using the microbroth dilution method, the antibacterial activities of **3** and **4** against MRSA, *Mycolicibacterium* (*Mycobacterium*) *smegmatis* and *Klebsiella pneumoniae* were determined (Figure S21). The MICs of these compounds were greater than 64 μg/mL, and no significant inhibitory activity was observed (Table 3).

### 2.5. Molecular Docking

To investigate the molecular interactions between **4** and α-glucosidase, a molecular docking study was performed using the program AutoDock Vina 1.1.2. The molecular docking models of **4** are illustrated in Figure 2. The docking results revealed that **4** formed a hydrogen bond and a hydrophobic interaction with Tyr-299, a hydrophobic interaction with Trp-406 and one salt bridge with the His-600 residue (Figure 2). Furthermore, the affinities of the aforementioned inhibitors were calculated, revealing that acarbose has a binding energy of 5.5 kcal/mol and that **4** has a binding energy of 5.4 kcal/mol; therefore, these compounds may stably bind to α-glucosidase (Table S1). The results obtained for the docking energy and α-glucosidase inhibitory activity experiments corresponded well. As shown in Figure S20, more hydrogen bonds and hydrophobic bonds formed between acarbose and α-glucosidase than between compound **4** and α-glucosidase. This docking experiment may provide insight into mechanisms by which α-pyrone and α-glucosidase bind since different intermolecular interactions may exert varying inhibitory effects.

### 2.6. Molecular Dynamics Simulations

Subsequently, a molecular dynamics simulation was run under physiologically simulated conditions to clarify the binding pattern, stability, and molecular interaction mode of **4** with the α-glucosidase protein complex. Root-mean-square deviation (RMSD), root-mean-square fluctuation (RMSF), and hydrogen bond studies were utilized to investigate the dynamic changes and stability of complex systems. Structural stability is often characterized by low RMSD and RMSF values [18]. As shown in Figure 3A, the RMSD of the two systems, α-glucosidase/acarbose and α-glucosidase/diaporpyrone D (**4**), are plotted in the RMSD variation graphs during the simulation. The two systems gradually converge in the first 5 ns of the simulation and maintain very stable fluctuations in the subsequent simulations, with the RMSD maintaining fluctuations within 1–2 Å. Based on the stable fluctuations of the two systems, the systems are stable in combination. As shown in Figure 3B, the RMSF of all proteins after binding different small molecules was low, which indicates that the core structure of the proteins has good rigidity. Therefore, these proteins are more rigid as binding small molecules, and these small molecules have an inhibitory effect. Notably, the red line and the blue line have a high degree of superposition, indicating that the two small molecules have similar effects on the proteins. The radius of gyration (RoG) reflects the embodied compactness and can reflect the degree of densification of the system. In Figure 3C, we can observe that α-glucosidase/acarbose and α-glucosidase/diaporpyrone D (**4**) fluctuate similarly, and both systems exhibit similar binding effects. A detailed analysis revealed that the RoG of α-glucosidase/acarbose was mostly smaller during the simulation, implying that the system became more compact, corresponding to relatively stronger binding.

Based on the trajectories of the molecular dynamic simulations, we calculated the binding energies using the MM-GBSA method, which can more accurately reflect the binding modes of small molecules and target proteins. The binding energies of the α-glucosidase/acarbose and α-glucosidase/diaporpyrone D (**4**) complexes were −36.59 ± 3.30 and −23.06 ± 3.77 kcal/mol, respectively (Table S2), and negative values indicate that the two molecules have the potential to bind to the target proteins, while lower values indicate stronger binding. The lower the value is, the stronger the binding. Our calculations show that compared to diaporpyrone D (**4**), α-glucosidase/acarbose binds better and has a value slightly lower. For the α-glucosidase/acarbose complex, the binding energy is mainly contributed by electrostatic energy and van der Waals energy; for α-glucosidase/diaporpyrone D (**4**), the binding energy is mainly contributed by van der Waals energy. The nonpolar solvation free energy contributes weakly to both complexes. Hydrogen bonding is among the strongest noncovalent binding interactions, and a greater number of hydrogen bonds indicates better binding. Figure 3D shows that the number of hydrogen bonds of α-glucosidase/acarbose was maintained at 2–9 and mostly fluctuated around 5, which implies that hydrogen bonding plays an important role in the stabilization of acarbose binding. In contrast, the number of hydrogen bonds in the α-glucosidase/diaporpyrone D (**4**) complex fluctuated more during the simulation period (0–5), and the number of hydrogen bonds was greater in the presimulation period (2–4); the number of hydrogen bonds was also lower in the middle of the simulation period (0–2) and late simulation period, (0–4). This finding implies that hydrogen bonding contributes weakly to α-glucosidase/diaporpyrone D (**4**) binding.

## 3. Materials and Methods

### 3.1. General Methods

As previously reported, various instruments (including those used for MS and NMR) and standard reagents for chemical isolation and biological evaluation were utilized [17]. The details are provided in the Supplementary Information.

### 3.2. Fungal Strain

In previous publications, we described *Diaporthe* sp. CB10100 in detail [17].

### 3.3. Fermentation of Diaporthe *sp.* CB10100

The fermentation and extraction processes for *Diaporthe* sp. CB10100 were identical to those reported in previous work [17].

### 3.4. Isolation of Compounds **3**–**4**

According to previous methods, the EtOAc-soluble fraction (192.8 g) was chromatographed on silica gel columns (200–300 mesh) to yield nine combined fractions (Fr. A to I) [17]. Fr.I (57.86 g) was separated on an ODS column (H_2_O/MeOH, *v*/*v* 8:2 → 7:3 → 6:4 → 5:5 → 4:6 → 3:7 → 2:8 → 1:9 → 0:1) to generate sixteen fractions (Fr.I-1 to Fr.I-16). Fr.I-1 (49.9991 g) was subsequently run through an ODS column (H_2_O/MeOH, *v*/*v* 98:2 → 95:5 → 90:10 → 85:15 → 80:20 → 70:30 → 60:40 → 50:50 → 40:60 → 30:70 → 20:80 → 10:90 → 0:100) to generate eighteen fractions (Fr.I-1-1 to Fr.I-1-18). The MCI GEL CHP20/P120 was chosen to separate Fr.I-1-3 (1.7794 g) from the mobile phase of H_2_O/MeOH (*v*/*v*, 98:2 → 95:5 → 90:10 → 85:15 → 80:20 → 70:30 → 60:40 → 50:50 → 40:60 → 30:70 → 20:80 → 10:90 → 0:100), and eight fractions were separated (Fr.I-1-3-1 to Fr.I-1-3-8). Fr.I-1-3-3 (0.0410 g) was purified using semipreparative HPLC with a gradient of MeCN/H_2_O [containing 0.03% formic acid, MeCN/H_2_O, *v*/*v*, 5:95 → 20:80 (0–10 min); 20:80 (10–12 min); 20:80 → 5:95 (12–12.5 min); 5:95 (12.5–20 min)] as the mobile phase to yield diaporpyrone F (**3**, 1.57 mg, 11.7 min) and diaporpyrone D (**4**, 1.70 mg, 15.7 min).

#### Diaporpyrone F (**3**)

Colorless gum; LC-UV (ACN/H_2_O/0.03% FA) λmax 193.5, 220.6, 306.0; ^1^H, ^13^C and 2D NMR spectroscopic data, see Table 1 and Figures S1–S10; HRESIMS *m*/*z* 183.06487 [M + H]^+^ (calcd for C_9_H_11_O_4_, 183.06519)

### 3.5. α-Glucosidase Inhibition Assay

The inhibitory activity of compounds **3** and **4** against α-glucosidase [Sigma-Aldrich (Shanghai) Trading Co., Ltd., Shanghai, China, Product No. G5003] was examined using the Worawalai technique with minor modifications [19]. The in vitro α-glucosidase inhibitory activity test was performed spectrophotometrically by detecting the α-glucosidase levels at 405 nm. The reaction system is described in the Supplementary Information.

### 3.6. Antibacterial Assay

The broth dilution technique was used to determine the MICs [20]. The specifics are included in the Supplementary Information.

### 3.7. Molecular Docking Analysis

The approach is outlined in the Supplementary Information [21,22,23].

### 3.8. Molecular Dynamic Simulations

The approach is described in the Supplementary Information [24,25,26,27,28,29,30,31,32,33].

## 4. Conclusions

In conclusion, a new α-pyrone, diaporpyrone F (**3**), together with one known compound, diaporpyrone D (**4**), was isolated from the endophytic fungus *Diaporthe* sp. CB10100. NMR and HRESIMS spectra were used to establish the structures of **3** and **4**. Bioinformatics analysis revealed that biosynthetic gene clusters for α-pyrone analogs are common in fungi of *Diaporthe* species. These compounds were evaluated for their inhibitory activity against α-glucosidase, and **4** showed a 46.40% inhibitory effect against α-glucosidase at 800 μM. Evaluations of the inhibitory activity against MRSA, *M. smegmatis* and *K. pneumoniae* revealed that the MICs of these compounds were greater than 64 μg/mL. Molecular docking and molecular dynamics simulations of **4** with α-glucosidase further suggested that **4** is a potential α-glucosidase inhibitor. In view of the above results, α-pyrone skeletons can be further investigated as lead compounds for α-glucoside inhibitors.

## Figures and Tables

**Table 1:** The ^1^H NMR (500 MHz) and ^13^C NMR (125 MHz) data of diaporpyrone F (**3**) in DMSO-*d*_6_ (*δ* in ppm, *J* in Hz).

Position	Diaporpyrone F (3)	
	*δ*_C_, Type	*δ*_H_ (*J* in Hz)
2	161.8, C	
3	112.4, CH	6.12 (d, *J =* 9.3 Hz)
4	148.1, CH	7.46 (d, *J* = 9.4 Hz)
5	114.7, C	
6	158.9, C	
1′	24.7, CH_2_	2.49 (m)
2′	34.8, CH_2_	2.33 (t, *J* = 7.4 Hz)
3′	174.6, C	
1″	17.1, CH_3_	2.20, s

**Table 2:** Distribution of α-pyrone biosynthetic gene clusters (BGCs) in the genus *Diaporthe.*

	α-Pyrone BGC		α-Pyrone BGC		α-Pyrone BGC
*Diaporthe* ampelina	**··**	*Diaporthe* citri	**·**	*Diaporthe* longicolla	**··**
*Diaporthe* amygdali	**··**	*Diaporthe* citriasiana	**·**	*Diaporthe* nobilis	**··**
*Diaporthe* aspalathi	**··**	*Diaporthe* citrichinensis	**·**	*Diaporthe* sp. DP-2020a	**··**
*Diaporthe* batatas	**··**	*Diaporthe* destruens	**·**	*Diaporthe* sp. HANT25	**····**
*Diaporthe* capsici	**··**	*Diaporthe* eres	**··**	*Diaporthe* sp. NJD1	**··**
*Diaporthe* caulivora	**·**	*Diaporthe* ilicicola	**··**	*Diaporthe* vexans	**··**

**·**: one biosynthetic gene cluster; **··**: two biosynthetic gene clusters; **····**: four biosynthetic gene clusters.

**Table 3:** Inhibition of α-glucosidase and antibacterial activity (MICs, μg/mL) of **3** and **4**.

Activity	α-Glucosidase/Bacteria	Acarbose/Levofloxacin	3	4
% inhibition (800 μM)	α-glucosidase	57.33	-	46.4
antibacterial activities (μg/mL)	MRSA	0.5	>64	>64
	*M. smegmatis*	1	>64	>64
	*K. pneumoniae*	2	>64	>64

-: No anti-α-glucosidase activity observed.

## Data Availability

Data generated in the process of this research are available in the Supplementary Materials.

## Supplementary Materials

The following supporting information can be downloaded at: https://www.mdpi.com/article/10.3390/molecules29081768/s1, Table S1: Logarithms of free binding energies (FBE, kcal/mol) of diaporpyrone D (**4**) and acarbose to the active cavities of α-glucosidase (PDB ID: 2QMJ) and targeting residues of the binding site located on the mobile flap; Table S2: Binding free energies and energy components predicted by MM/GBSA (kcal/mol); Figure S1: ^1^H NMR spectrum of **3** in DMSO-*d*_6_ (500 MHz); Figure S2: ^13^C NMR spectrum of **3** in DMSO-*d*_6_ (125 MHz); Figure S3: DEPT-90 spectrum of **3**; Figure S4: DEPT-135 spectrum of **3**; Figure S5: HSQC spectrum of **3**; Figure S6: HMBC spectrum of **3**; Figure S7: ^1^H-^1^H COSY spectrum of **3**; Figure S8: NOESY spectrum of **3**; Figure S9: HRESIMS spectrum of **3**; Figure S10: UV spectrum of **3**; Figure S11: ^1^H NMR spectrum of **4** in DMSO-*d*_6_ (600 MHz); Figure S12: ^13^C NMR spectrum of **4** in DMSO-*d*_6_ (150 MHz); Figure S13: DEPT-135 spectrum of **4;** Figure S14: HSQC spectrum of **4**; Figure S15: HMBC spectrum of **4**; Figure S16: ^1^H-^1^H COSY spectrum of **4**; Figure S17: HRESIMS spectrum of **4**; Figure S18: UV spectrum of **4**; Figure S19: Structures of diaporpyrones A-D (**S1**–**S4**) isolated from endophilic fungus strain *Diaporthe* sp. CB10100; Figure S20: Docking poses and interactions of acarbose with α-glucosidase (PDB ID: 2QMJ); Figure S21: 96 well plate assay of **3**–**4** against MRSA (**A**), *Mycolicibacterium* (*Mycobacterium*) *smegmatis* (**B**) and *Klebsiella pneumonia* (**C**) using the microbroth dilution method.

## References

[1] Su J., Tang L., Luo Y., Xu J., Ouyang S. (2023). Research Progress on Drugs for Diabetes Based on Insulin Receptor/Insulin Receptor Substrate. Biochem. Pharmacol..

[2] Saeedi P., Petersohn I., Salpea P., Malanda B., Karuranga S., Unwin N., Colagiuri S., Guariguata L., Motala A.A., Ogurtsova K. (2019). Global and Regional Diabetes Prevalence Estimates for 2019 and Projections for 2030 and 2045: Results from the International Diabetes Federation Diabetes Atlas, 9th Edition. Diabetes Res. Clin. Pract..

[3] Majety P., Lozada Orquera F.A., Edem D., Hamdy O. (2023). Pharmacological Approaches to the Prevention of Type 2 Diabetes Mellitus. Front. Endocrinol..

[4] Blahova J., Martiniakova M., Babikova M., Kovacova V., Mondockova V., Omelka R. (2021). Pharmaceutical Drugs and Natural Therapeutic Products for the Treatment of Type 2 Diabetes Mellitus. Pharmaceuticals.

[5] Sohretoglu D., Renda G., Arroo R., Xiao J., Sari S. (2023). Advances in the Natural α-Glucosidase Inhibitors. eFood.

[6] Agrawal S., Bhatt A. (2023). Microbial Endophytes: Emerging Trends and Biotechnological Applications. Curr. Microbiol..

[7] Wang Z., Wang L., Pan Y., Zheng X., Liang X., Sheng L., Zhang D., Sun Q., Wang Q. (2023). Research Advances on Endophytic Fungi and Their Bioactive Metabolites. Bioprocess. Biosyst. Eng..

[8] Kandasamy G.D., Kathirvel P. (2023). Insights into Bacterial Endophytic Diversity and Isolation with a Focus on Their Potential Applications -A Review. Microbiol. Res..

[9] Zhou Z.Y., Liu X., Cui J.L., Wang J.H., Wang M.L., Zhang G. (2022). Endophytic Fungi and Their Bioactive Secondary Metabolites in Medicinal Leguminosae Plants: Nearly Untapped Medical Resources. FEMS Microbiol. Lett..

[10] Jiang L., Ma Q., Li A., Sun R., Tang G., Huang X., Pu H. (2023). Bioactive Secondary Metabolites Produced by Fungi of the Genus *Diaporthe* (*Phomopsis*): Structures, Biological Activities, and Biosynthesis. Arab. J. Chem..

[11] Chang J.L., Pei J., Zhou Y.H., Ouyang Q.X., Qin C.L., Hu J.Y., Meng X.G., Ruan H.L. (2024). Diaporaustalides A–L, Austalide Meroterpenoids from a Plant Endophytic *Diaporthe* sp.. J. Nat. Prod..

[12] Khan B., Li Y., Wei W., Liu G., Xiao C., He B., Zhang C., Rajput N.A., Ye Y., Yan W. (2023). Chemical Investigation of Endophytic *Diaporthe Unshiuensis* YSP3 Reveals New Antibacterial and Cytotoxic Agents. J. Fungi.

[13] Martínez-Aldino I.Y., Rivera-Chávez J., Morales-Jiménez J. (2023). Integrating Taxonomic and Chemical Diversity of Mangrove-Associated Ascomycetes to Discover or Repurpose Bioactive Natural Products. J. Nat. Prod..

[14] McGlacken G.P., Fairlamb I.J.S. (2005). 2-Pyrone Natural Products and Mimetics: Isolation, Characterisation and Biological Activity. Nat. Prod. Rep..

[15] Zhang Y., Liu H., Chen Y., Wei S., Zhang W., Tan H. (2022). Cytospones E-J from the Endophytic Fungus *Cytospora Rhizophorae*. Fitoterapia.

[16] Zhang J., Zhang B., Cai L., Liu L. (2022). New Dibenzo-α-Pyrone Derivatives with α-Glucosidase Inhibitory Activities from the Marine-Derived Fungus *Alternaria Alternata*. Mar. Drugs.

[17] Pu H., Liu J., Wang Y., Peng Y., Zheng W., Tang Y., Hui B., Nie C., Huang X., Duan Y. (2021). Bioactive α-Pyrone Derivatives from the Endophytic Fungus *Diaporthe* sp. CB10100 as Inducible Nitric Oxide Synthase Inhibitors. Front. Chem..

[18] Ye C., Zhang R., Dong L., Chi J., Huang F., Dong L., Zhang M., Jia X. (2022). α-Glucosidase Inhibitors from Brown Rice Bound Phenolics Extracts (BRBPE): Identification and Mechanism. Food Chem..

[19] Xu X.T., Deng X.Y., Chen J., Liang Q.M., Zhang K., Li D.L., Wu P.P., Zheng X., Zhou R.P., Jiang Z.Y. (2020). Synthesis and Biological Evaluation of Coumarin Derivatives as α-Glucosidase Inhibitors. Eur. J. Med. Chem..

[20] Wiegand I., Hilpert K., Hancock R.E.W. (2008). Agar and Broth Dilution Methods to Determine the Minimal Inhibitory Concentration (MIC) of Antimicrobial Substances. Nat. Protoc..

[21] Trott O., Olson A.J. (2010). AutoDock Vina: Improving the Speed and Accuracy of Docking with a New Scoring Function, Efficient Optimization, and Multithreading. J. Comput. Chem..

[22] Sanner M.F. (1999). Python: A Programming Language for Software Integration and Development. J. Mol. Graph. Model..

[23] Morris G.M., Huey R., Lindstrom W., Sanner M.F., Belew R.K., Goodsell D.S., Olson A.J. (2009). AutoDock4 and AutoDockTools4: Automated Docking with Selective Receptor Flexibility. J. Comput. Chem..

[24] Salomon-Ferrer R., Case D.A., Walker R.C. (2013). An Overview of the Amber Biomolecular Simulation Package. WIREs Comput. Mol. Sci..

[25] Sagui C., Darden T.A. (1999). Molecular Dynamics Simulations of Biomolecules: Long-Range Electrostatic Effects. Annu. Rev. Biophys. Biomolec. Struct..

[26] Kräutler V., Van Gunsteren W.F., Hünenberger P.H. (2001). A Fast SHAKE Algorithm to Solve Distance Constraint Equations for Small Molecules in Molecular Dynamics Simulations. J. Comput. Chem..

[27] Larini L., Mannella R., Leporini D. (2007). Langevin Stabilization of Molecular-Dynamics Simulations of Polymers by Means of Quasisymplectic Algorithms. J. Chem. Phys..

[28] Hou T., Wang J., Li Y., Wang W. (2011). Assessing the Performance of the MM/PBSA and MM/GBSA Methods. 1. The Accuracy of Binding Free Energy Calculations Based on Molecular Dynamics Simulations. J. Chem. Inf. Model..

[29] Chen Y., Zheng Y., Fong P., Mao S., Wang Q. (2020). The Application of the MM/GBSA Method in the Binding Pose Prediction of FGFR Inhibitors. Phys. Chem. Chem. Phys..

[30] Genheden S., Ryde U. (2015). The MM/PBSA and MM/GBSA Methods to Estimate Ligand-Binding Affinities. Expert Opin. Drug Discov..

[31] Rastelli G., Del Rio A., Degliesposti G., Sgobba M. (2010). Fast and Accurate Predictions of Binding Free Energies Using MM-PBSA and MM-GBSA. J. Comput. Chem..

[32] Nguyen H., Roe D.R., Simmerling C. (2013). Improved Generalized Born Solvent Model Parameters for Protein Simulations. J. Chem. Theory Comput..

[33] Weiser J., Shenkin P.S., Still W.C. (1999). Approximate Atomic Surfaces from Linear Combinations of Pairwise Overlaps (LCPO). J. Comput. Chem..

