# Supplementary material for: Bioactive α-Pyrone Analogs from the Endophytic Fungus Diaporthe sp. CB10100: α-Glucosidase Inhibitory Activity, Molecular Docking, and Molecular Dynamics Studies
Source: Molecules. 2024 Apr 12;29(8):1768. doi: 10.3390/molecules29081768 (PMC11052008; doi:10.3390/molecules29081768)
Supplement: Supplementary file 1 [file molecules-29-01768-s001.zip › molecules-2935728-supplementary.pdf]

Supplementary Information

Bioactive α-Pyrone Analogs from the Endophytic Fungus *Diaporthe* sp. CB10100: α-Glucosidase Inhibitory Activity, Molecular Docking, and Molecular Dynamics Studies

Zhong Wang[1,†], Qingxian Ma[1,†], Guangling Wu[1], Yani Zhong[1], Bin Feng[2], Pingzhi Huang[1], Aijie Li[1], Genyun Tang[1], Xueshuang Huang[1,*], and Hong Pu[1,*]

[1] Hunan Provincial Key Laboratory for Synthetic Biology of Traditional Chinese Medicine, School of Pharmaceutical Sciences, Hunan University of Medicine, Huaihua 418000, China; 13773980672@163.com (Z.W.); 17608470326@163.com (Q.M.); guangling2024@126.com (G.W.); 18874880070@163.com (Y.Z.); 19918037381@163.com (P.H.); 15274565209@163.com (A.L.); tanggenyun@foxmail.com (G.T.)

[2] Huaihua Hospital of Traditional Chinese Medicine, Huaihua 418000, China; txz-1221@163.com (B.F.)

* Correspondence: xueshuanghuang@126.com (X.H.); ph0745@126.com (H.P.)

† These authors contributed equally to this work.

Table of Contents

Experimental Procedures 3
General experimental procedures 3
α-Glucosidase inhibition assay 3
Antibacterial assay 4
Molecular docking 4
Molecular dynamic simulations 5
MM/GBSA binding free energy calculation 5
Table S1 Logarithms of free binding energies (FBE, kcal/mol) of diaporpyrone D (**4**) and acarbose to the active cavities of *α*-glucosidase (PDB ID: 2QMJ) and targeting residues of the binding site located on the mobile flap. 6
Table S2 Binding free energies and energy components predicted by MM/GBSA (kcal/mol) 7
Figure S1 ^{1}H NMR spectrum of **3** in DMSO-d_6 (500 MHz) 8
Figure S2 ^{13}C NMR spectrum of **3** in DMSO-d_6 (125 MHz) 9
Figure S3 DEPT-90 spectrum of **3** 10
Figure S4 DEPT-135 spectrum of **3** 11
Figure S5 HSQC spectrum of **3** 12
Figure S6 HMBC spectrum of **3** 13
Figure S7 ^{1}H-^{1}H COSY spectrum of **3** 14
Figure S8 NOESY spectrum of **3** 15
Figure S9 HRESIMS spectrum of **3** 16
Figure S10 UV spectrum of **3** 17
Figure S11 ^{1}H NMR spectrum of **4** in DMSO-d_6 (600 MHz) 18
Figure S12 ^{13}C NMR spectrum of **4** in DMSO-d_6 (150 MHz) 19
Figure S13 DEPT-135 spectrum of **4** 20
Figure S14 HSQC spectrum of **4** 21
Figure S15 HMBC spectrum of **4** 22
Figure S16 ^{1}H-^{1}H COSY spectrum of **4** 23
Figure S17 HRESIMS spectrum of **4** 24
Figure S18 UV spectrum of **4** 25
Figure S19 Structures of diaporpyrones A-D (**S1-S4**) isolated from endophilic fungus strain *Diaporthe* sp. CB10100. 26
Figure S20 Docking poses and interactions of acarbose with *α*-glucosidase (PDB ID: 2QMJ). 27
Figure S21 96 well plate assay of **3-4** against MRSA (**A**), *Mycolicibacterium (Mycobacterium) smegmatis* (**B**) and *Klebsiella pneumonia* (**C**) using the microbroth dilution method. 28
References 29

Experimental Procedures

General experimental procedures

UV spectra were measured on a Waters 2998 PDA Detector [Waters Technology (Shanghai) Co., Ltd., Shanghai, China]. Semi-preparative reversed-phase high performance liquid chromatography (RP-HPLC) was performed using a Waters 1525 Binary HPLC pump equipped with a Waters 2489 UV/visible detector [Waters Technology (Shanghai) Co., Ltd., Shanghai, China] and using a Welch Ultimate AQ-C18 column (250 × 10 mm, 5 μm) [Welch Technology (shanghai) Co., Ltd., Shanghai, China]. HRESIMS spectra were recorded on a Q-Exactive Focus Orbitrap MS (Thermo Electron, Bremen, Germany) connected to the Thermo Scientific Dionex Ultimate 3000 RS (Thermo Fisher Scientific, California, USA). NMR spectra were acquired using a Bruker 500 MHz or 600 MHz spectrometer (Bruker Corporation, Massachusetts, USA). The Chemical shifts in ^{1}H NMR and ^{13}C NMR spectra were referenced to the solvents for DMSO-d_6 (δ_H 2.50 and δ_C 39.6) (Cambridge Isotope Laboratories, Inc., Massachusetts, USA). Column chromatography (CC) was carried out on silica gel (200–300 mesh, Yantai Jiangyou Silica Gel Development Co., Ltd., Yantai, China), RP-C18 (AAG12S50, YMC Co., Ltd., Kyoto, Japan), MCI GEL CHP20/P120 (Mitsubishi Chemical Group Corporation, Tokyo, Japan) .

α-Glucosidase inhibition assay

The inhibitory activity of compounds **3** and **4** against *α*-glucosidase [Sigma-Aldrich (Shanghai) Trading Co., Ltd., Shanghai, China, Product No. G5003] was investigated according to the method of Worawalai with a slight modification[1]. Those compounds were dissolved in DMSO (Shanghai Macklin Biochemical Technology Co., Ltd., Shanghai, China). *α*-glucosidase enzyme and 4-Nitrophenyl *α*-D-glucopyranoside (PNPG) (Shanghai Aladdin Biochemical Technology Co., Ltd., Shanghai, China) were dissolved in 0.1 M potassium phosphate buffer (Shanghai Aladdin Biochemical Technology Co., Ltd., Shanghai, China) (pH 6.8) independently. Four groups were set up, namely the test group with the test compound and the enzyme, control group with only the enzyme, sample group with only the test compound, and blank group without anything, while every group was added with potassium phosphate buffer and PNPG to build a reaction system of 200 μL. In the test group, 10 μL of *α*-glucosidase (final concentration 0.1 U/mL), 130 μL of phosphate buffer and 10 μL of the test compounds were added into 96 well plates (Corning Incorporated, New York, USA), in succession. After incubated at 37 ℃ for 20 min, 50 μL of PNPG (final concentration 1 mmol/L) was added into the mixture, followed by incubation at 37 ℃ for 40 min. Finally, 100 μL of Na_2CO_3 (0.5 M) (Shanghai Aladdin Biochemical

Technology Co., Ltd., Shanghai, China) was added into the mixture to stop reaction. The enzymatic activity was quantified by measuring the absorbance at 405 nm using multi-mode microplate reader (BMG Labtech, Ortenberg, Germany). The inhibition percentage was calculated as follows: % of inhibition = $[1-(A_s-A_t)/(A_c-A_b)] \times 100\%$, where A_s is the absorbance of the sample group; At is the absorbance of the test group; Ac is the absorbance of the control group; Ab is the absorbance of the blank group.

Antibacterial assay

Compounds **3**-**4** were tested for antibacterial activity against methicillin-resistant *Staphylococcus aureus* (MRSA), *Mycolicibacterium (Mycobacterium) smegmatis* and *Klebsiella pneumonia*. The MICs were determined using broth dilution assay. The bacterial strains were cultured overnight and diluted to 10^6 CFU/mL in Luria-Bertani (LB) broth [yeast extract (Thermo Scientific Oxoid, Massachusetts, USA), tryptone (Thermo Scientific Oxoid, Massachusetts, USA), NaCl (Sinopharm Chemical Reagent Co., Ltd., Shanghai, China)]. **3**-**4** were dissolved in DMSO, serially diluted to 10 different concentrations (64, 32, 16, 8, 4, 2, 1, 0.5. 0.25, 0.125 μg/mL) using LB broth on the 96 well plate. Levofloxacin (Bide Pharmatech Ltd., Shanghai, China) was dissolved in DMSO, serially diluted to 10 concentrations (0.125–64 μg/mL) using LB broth on each 96 well plate. Then 100 μL LB broth containing compounds **3**-**4** or levofloxacin and 100 μL of bacterial solution were mixed per well. The plates were incubated at 37 °C for 18 h. Finally, 50 μL resazurin (Shanghai Macklin Biochemical Technology Co., Ltd., Shanghai, China) was added into each well to visualize the result. **3**-**4** and levofloxacin were tested in duplicate on each 96 well plate.

Molecular docking

Molecular docking studies were performed to investigate the binding mechanism between the derivative diaporpyrone D (**4**) and the N-terminal subunit of Human Maltase-Glucoamylase in Complex with acarbose (PDB ID: 2QMJ) using Autodock vina 1.1.2 [2]. The AutoDockTools 1.5.6 package[3,4] had been used for generating the docking input documents. The search grid of a-glucosidase was identified as center_x: -20.21, center_y: -6.763, and center_z: -9.383 with dimensions size_x: 15, size_y: 15, and size_z: 15. Vina docking generally used default parameters unless otherwise indicated. The optimal scoring pose was determined by the Vina docking score and PyMOL 1.8 software (https://www.pymol.org) was utilized to make visual analysis.

Molecular dynamic simulations

Molecular dynamics simulations were performed using AMBER 18 software for simulation [5]. Before the simulation, the system was energy optimized, including the steepest descent method with 2500 steps and the conjugate gradient method with 2500 steps. After the energy optimization of the system was completed, the temperature of the system was slowly increased from 0 K to 298.15 K by heating the system for 200 ps at a fixed volume and constant heating speed. Under the condition of the system maintenance temperature of 298.15 K, the NVT (isothermal isobody) system simulation was performed for 500 ps, so that the solvent molecules were further evenly distributed in the solvent box. Finally, the equilibrium simulation of the whole system was carried out for 500 ps in the case of NPT (isothermal and isopressure). Finally, the NPT (isothermal and isopressure) system simulation was carried out for 100 ns under periodic boundary conditions for the two composite systems. For simulations, the nonbonded cutoff distance was set to 10 Å, the Particle mesh Ewald (PME) method was used to calculate long-range electrostatic interactions[6], the SHAKE method was used to limit the length of hydrogen atomic bonds[7], and the Langevin algorithm was used for temperature control[8], where the collision frequency γ was set to 2 ps^{-1}. The system pressure was 1 atm, the integration step was 2 fs, and the trajectories were saved at 10 ps intervals for subsequent analysis.

MM/GBSA binding free energy calculation

The binding free energies between protein and ligand for all systems were calculated by the MM/GBSA method [9–12]. Long time molecular dynamics simulation may not be conducive to the accuracy of MM/GBSA calculation[9], so in this study, the MD trajectory of 45-50 ns is used for calculation, and the specific formula is as follows:

$$\Delta G_{bind} = \Delta G_{\mathrm{complex}} - (\Delta G_{\mathrm{receptor}} + \Delta G_{\mathrm{ligand}})$$
$$= \Delta E_{\mathrm{internal}} + \Delta E_{\mathrm{VDW}} + \Delta E_{\mathrm{elec}} + \Delta G_{\mathrm{GB}} + \Delta G_{\mathrm{SA}} \quad (1)$$

In equation (1), denotes the internal energy, denotes the van der Waals action and denotes the electrostatic interaction. The internal energy includes the bond energy (Ebond), the Angle energy (Eangle) and the torsion energy (Etorsion). And are collectively referred to as solvation free energies. Here, GGB is the polar solvation free energy and GSA is the non-polar solvation free energy. For, this paper uses the GB model developed by Nguyen et al. [13]for calculation (igb = 2). The non-polar solvation free energy (GSA) was calculated based on the product of the surface tension (γ) and the solvent accessible surface area (SA), GSA= 0.0072 × SASA[14]. Entropy change is ignored in this study due to high computational resource consumption and low accuracy[9,10].

Table S1 Logarithms of free binding energies (FBE, kcal/mol) of diaporpyrone D (**4**) and acarbose to the active cavities of α-glucosidase (PDB ID: 2QMJ) and targeting residues of the binding site located on the mobile flap.

compound	-log (FBE)	targeting residues
Diaporpyrone D	-5.4	TYR-299, TRP-406, HIS-600
Acarbose	-5.5	TYR-299, ASP-443, ASP327, ARG-526, TRP-406, GLN-603

Table S2 Binding free energies and energy components predicted by MM/GBSA (kcal/mol).

System	α-glucosidase/Acarbose	α-glucosidase/Diaporpyrone_D
ΔE_{vdW}	-30.55±2.59	-21.49±2.31
ΔE_{elec}	-513.80±8.88	232.65±7.13
ΔG_{GB}	513.98±8.70	-230.34±4.35
ΔG_{SA}	-6.22±0.18	-3.87±0.05
ΔG_{bind}	-36.59±3.30	-23.06±3.77

ΔE_{vdW}: van der Waals energy.

ΔE_{elec}: electrostatic energy.

ΔG_{GB}: electrostatic contribution to solvation.

ΔG_{SA}: non-polar contribution to solvation.

ΔG_{bind}: binding free energy.

Figure S1 ^{1}H NMR spectrum of **3** in DMSO-d_6 (500 MHz)

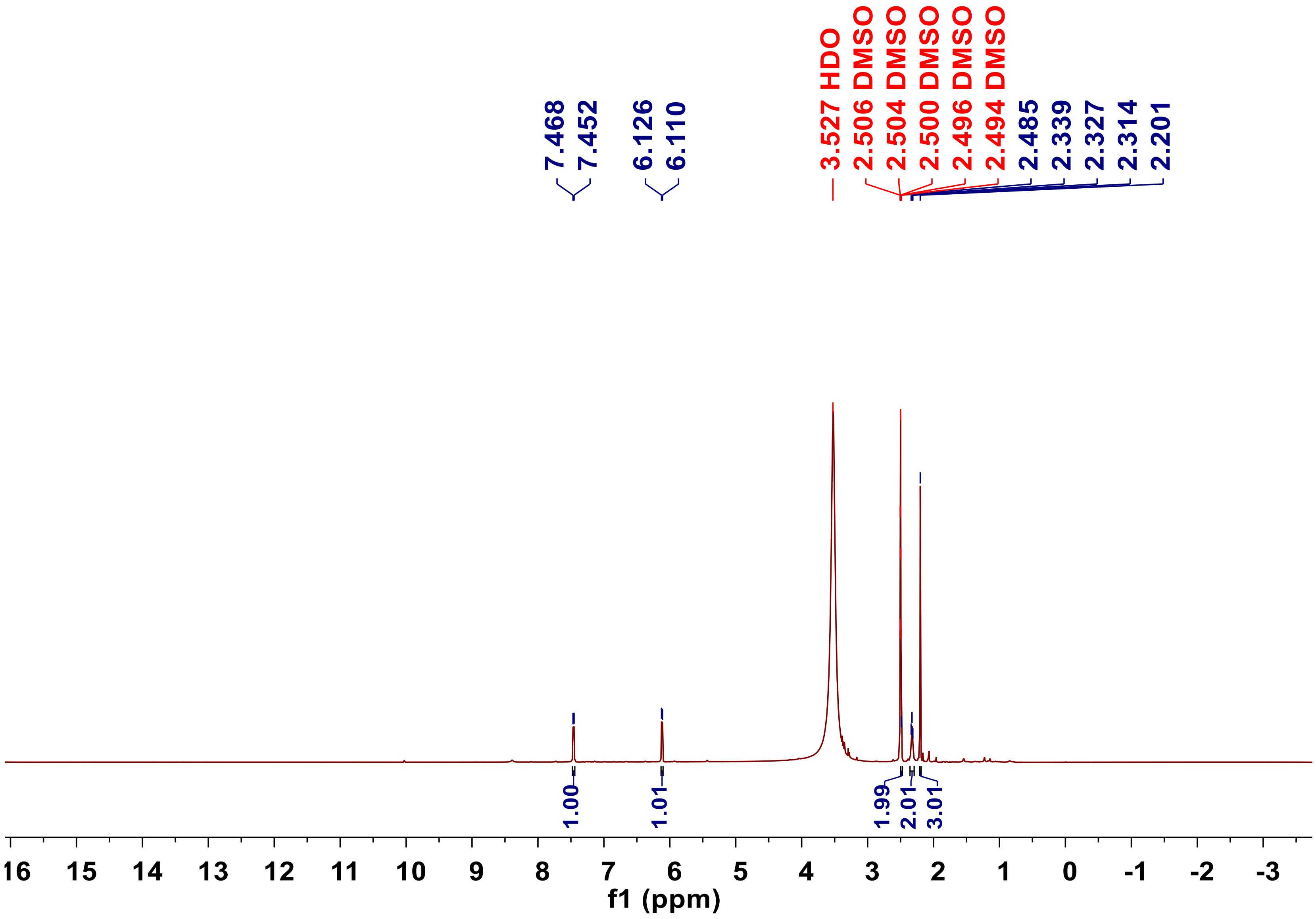

Figure S2 ^{13}C NMR spectrum of **3** in DMSO-d_6 (125 MHz)

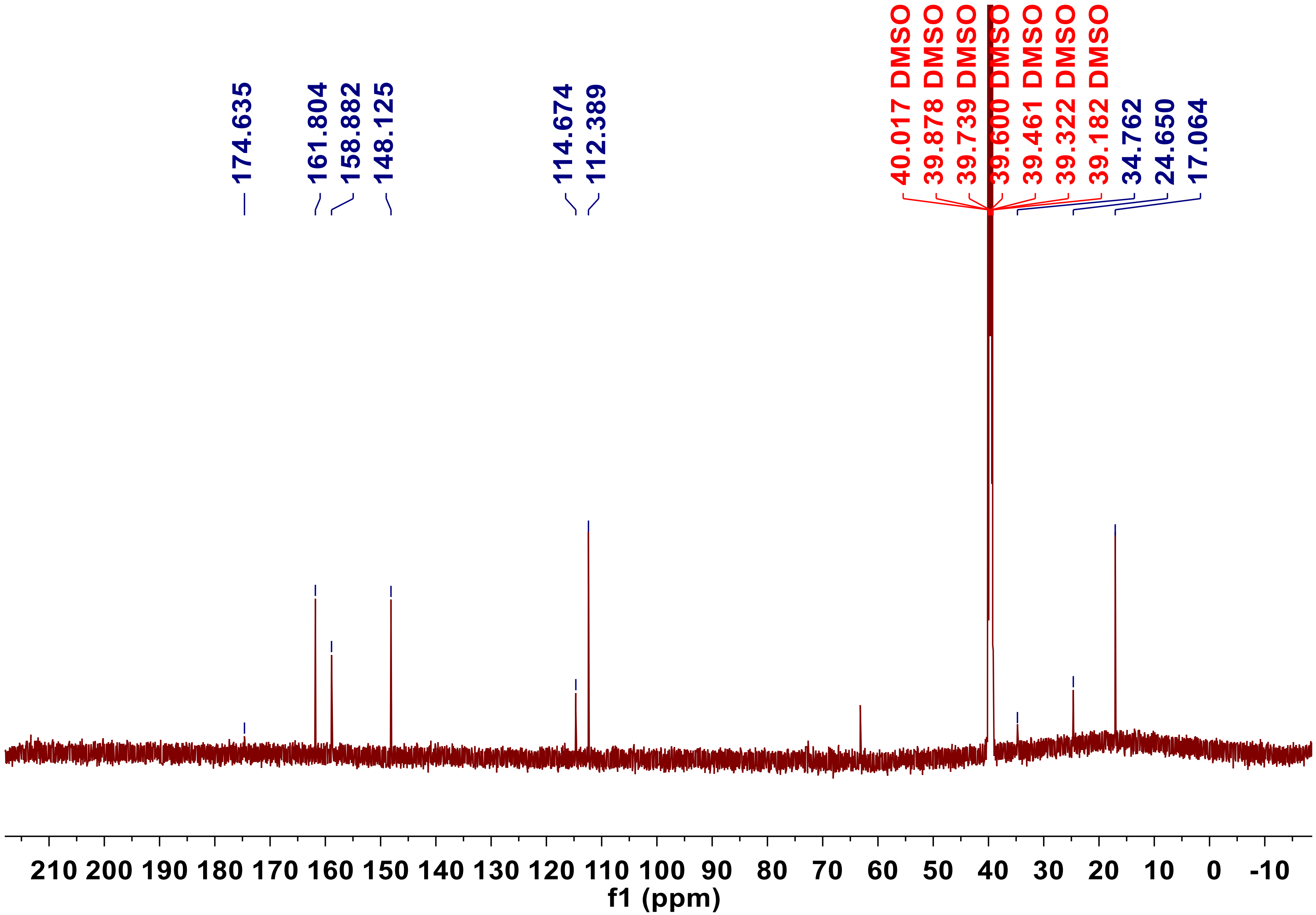

Figure S3 DEPT-90 spectrum of **3**

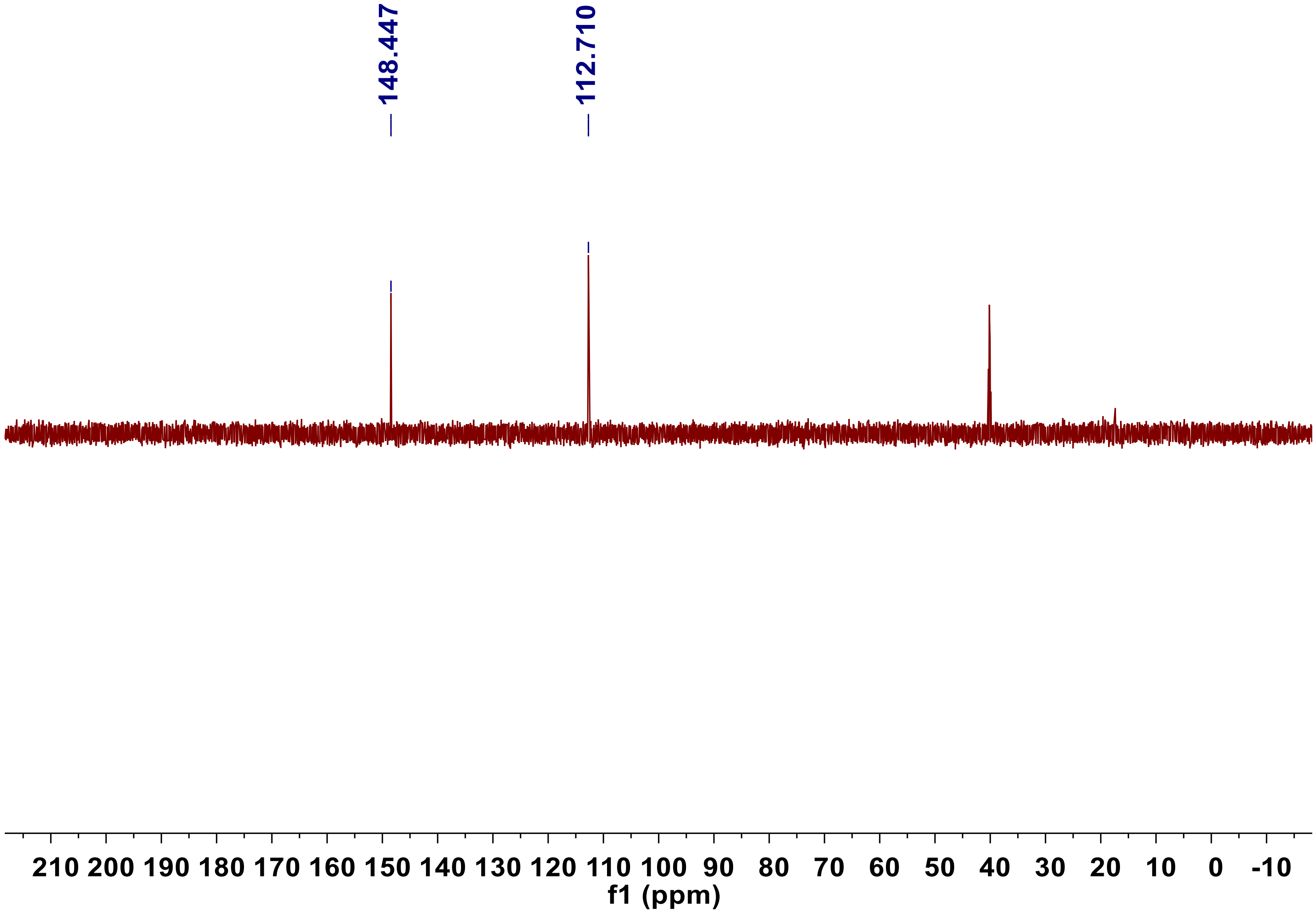

Figure S4 DEPT-135 spectrum of **3**

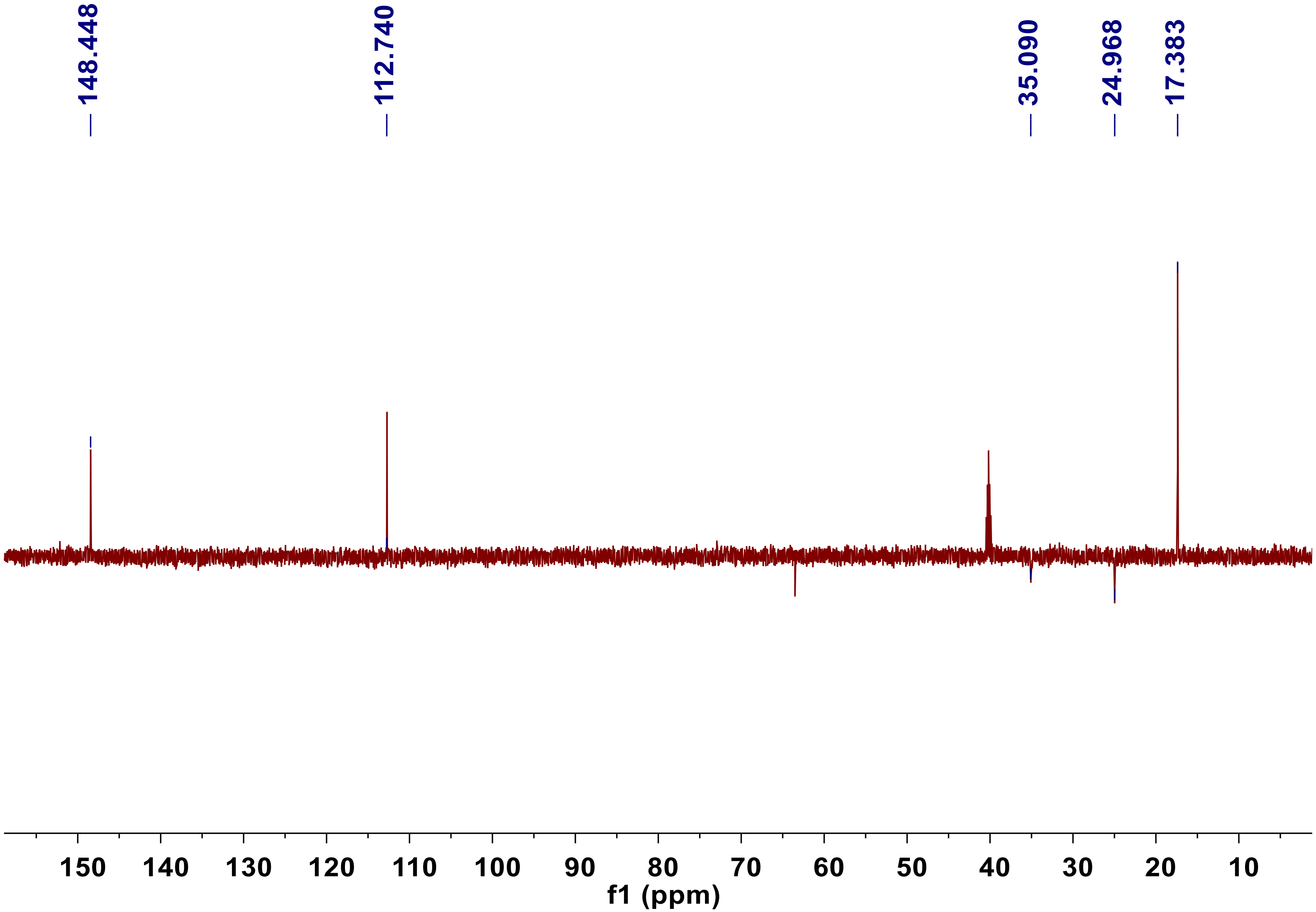

Figure S5 HSQC spectrum of **3**

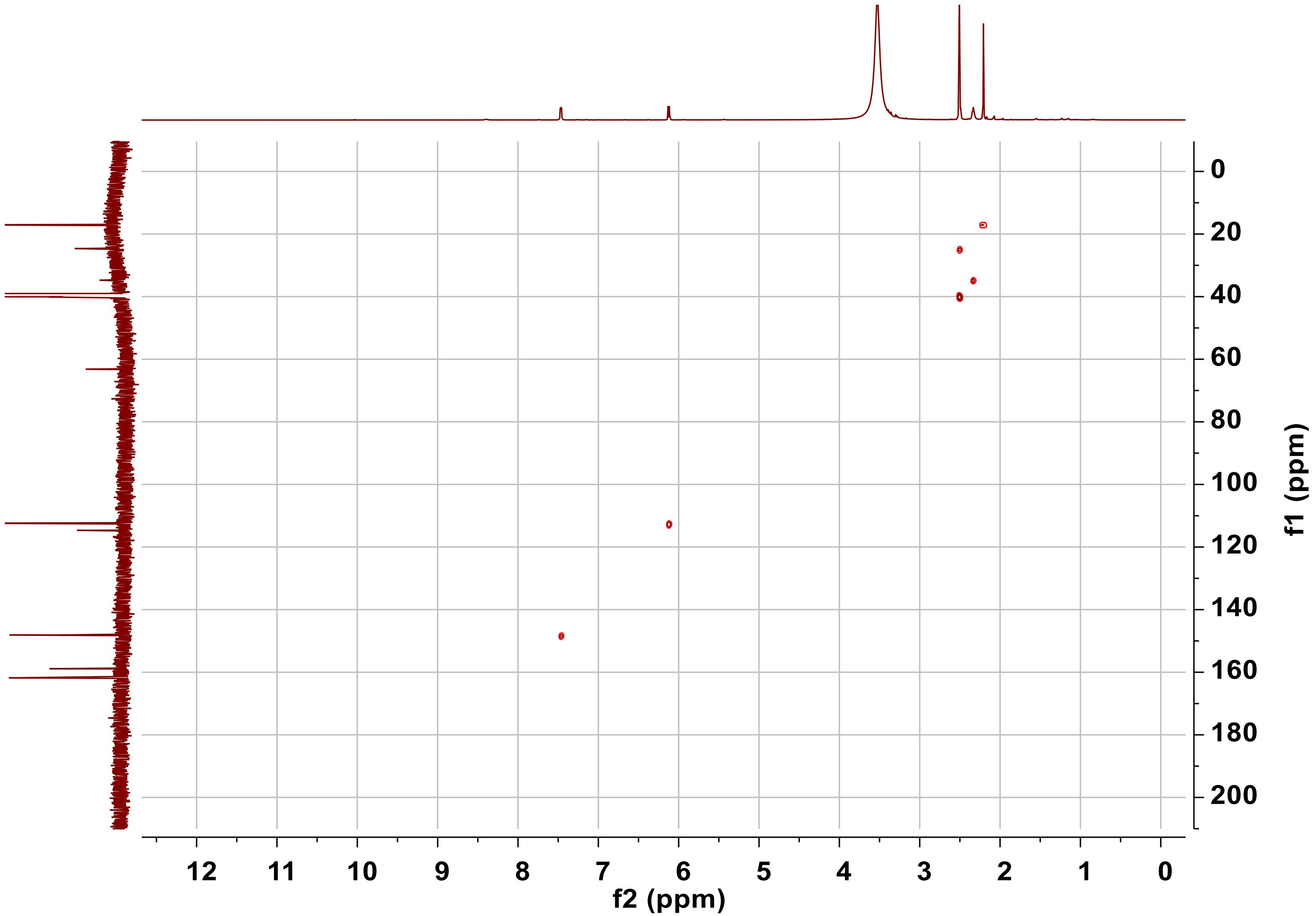

Figure S6 HMBC spectrum of **3**

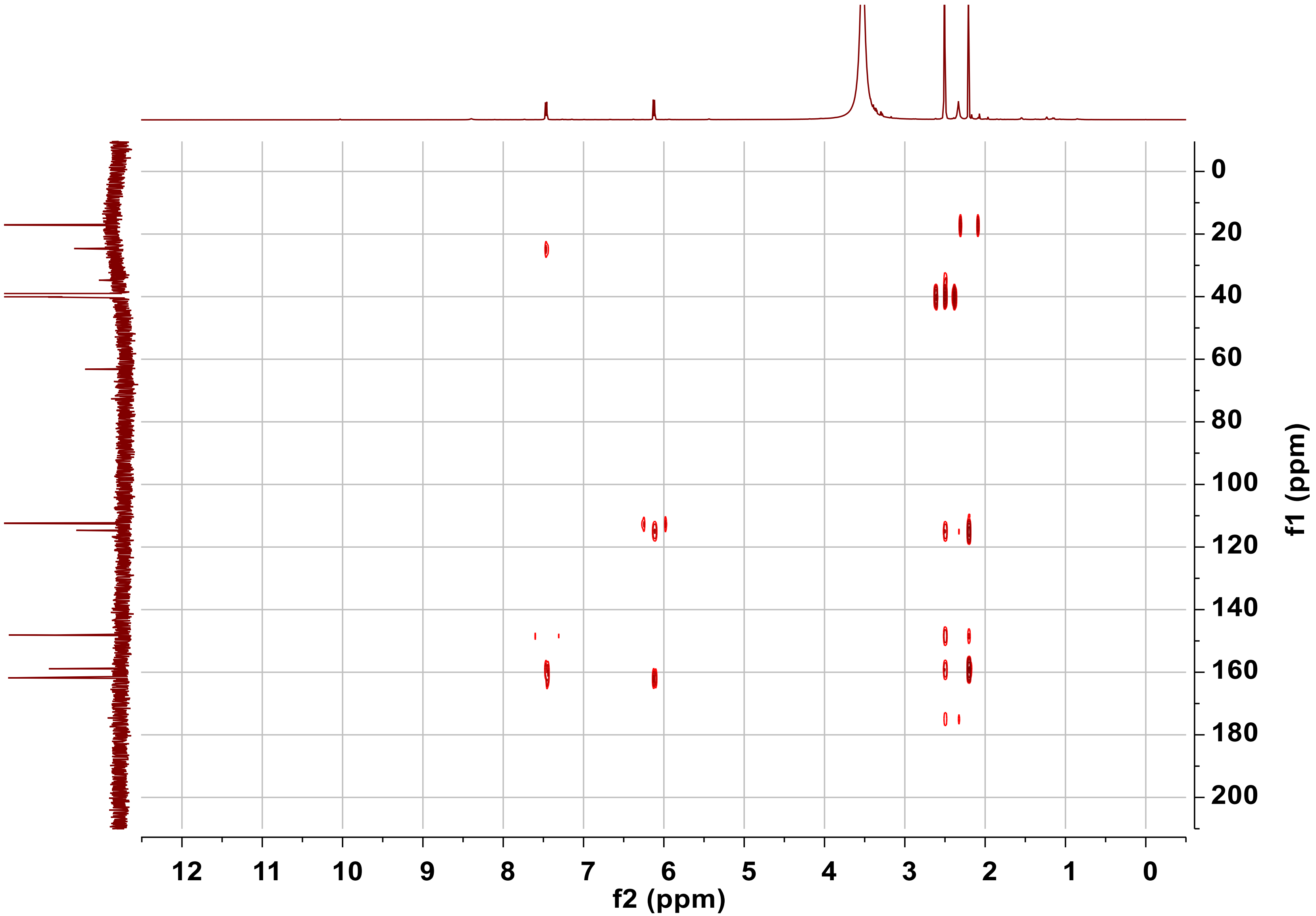

Figure S7 ^{1}H-^{1}H COSY spectrum of **3**

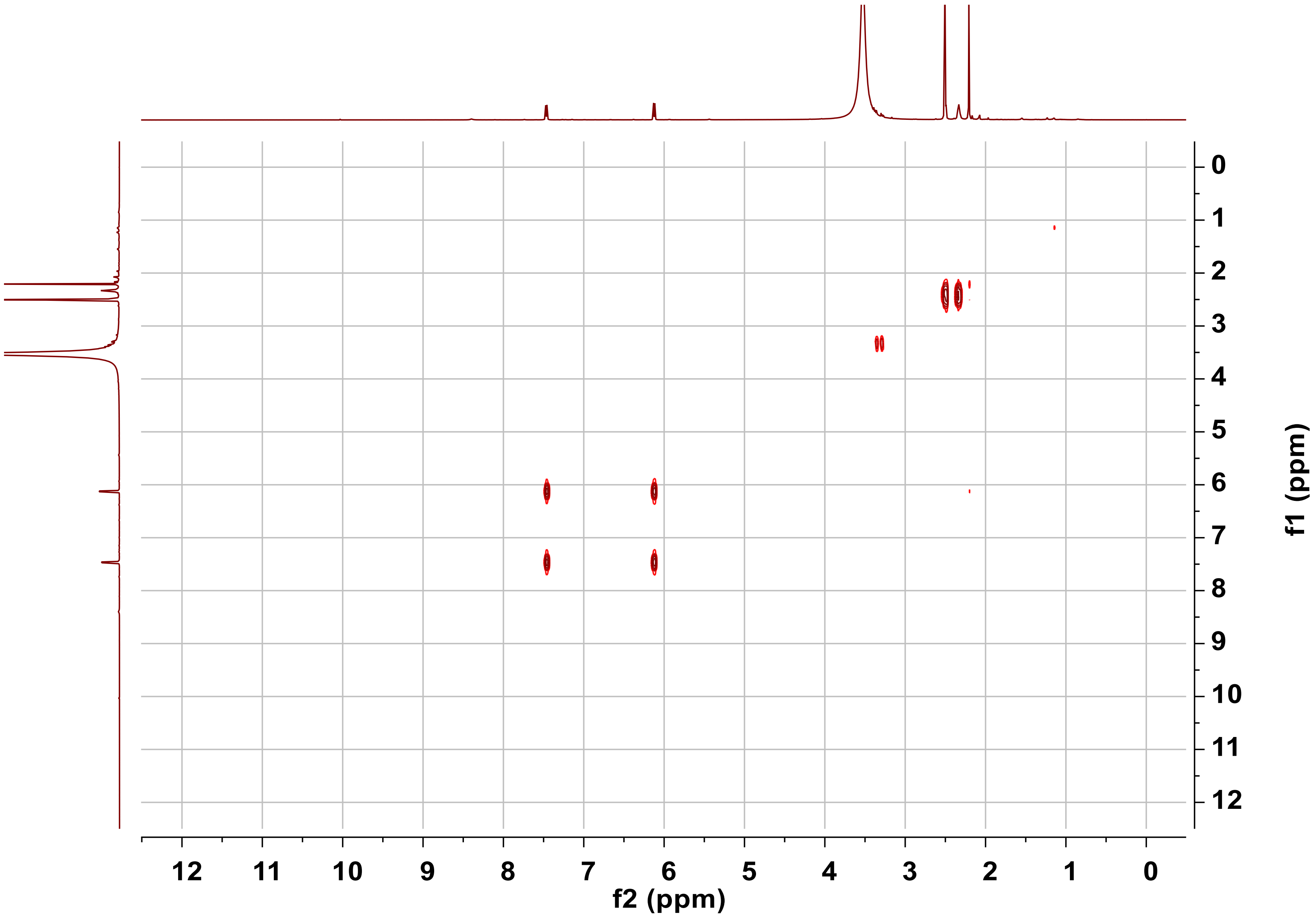

Figure S8 NOESY spectrum of **3**

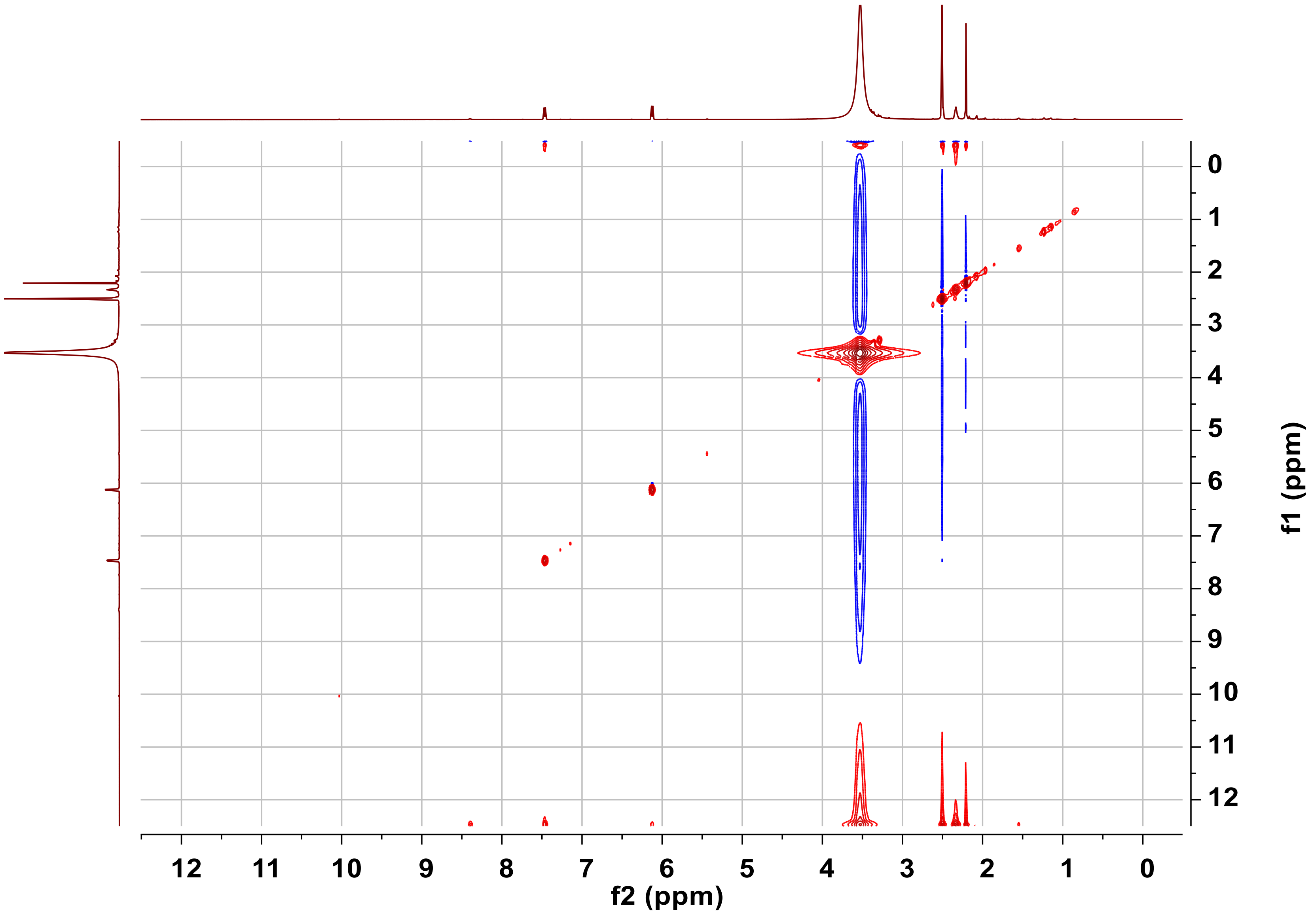

Figure S9 HRESIMS spectrum of **3**

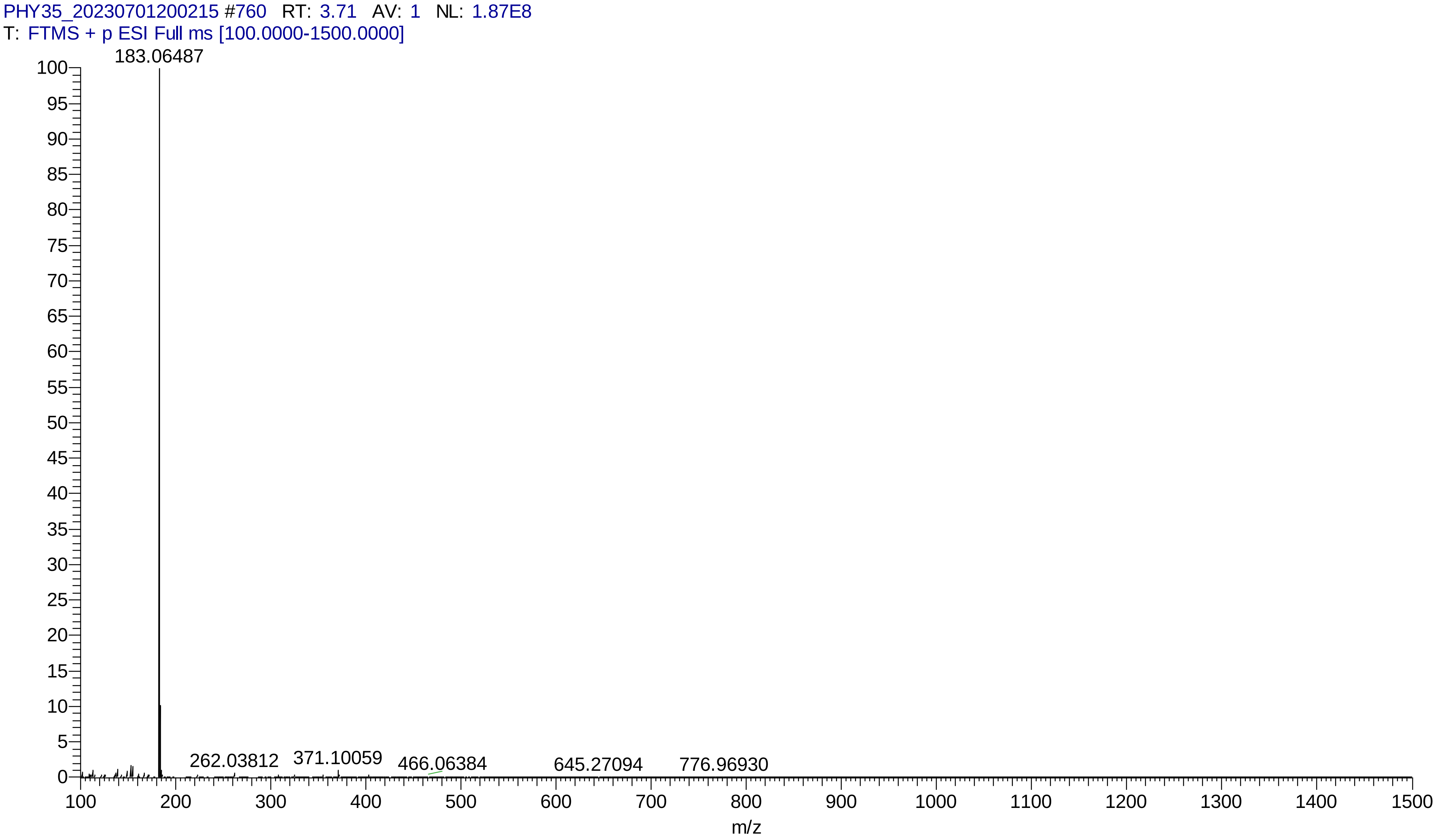

Figure S10 UV spectrum of **3**

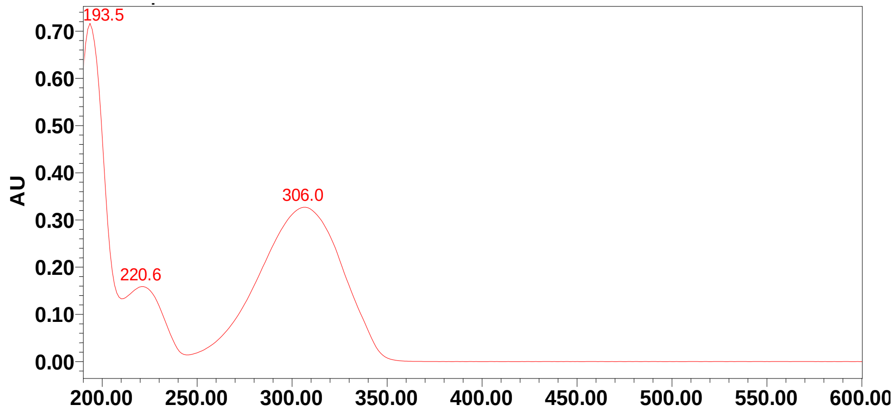

No.	Wavelength (nm)	Abs
1	193.5	0.717
2	220.6	0.159
3	306.0	0.327

Figure S11 ^{1}H NMR spectrum of **4** in DMSO-d_6 (600 MHz)

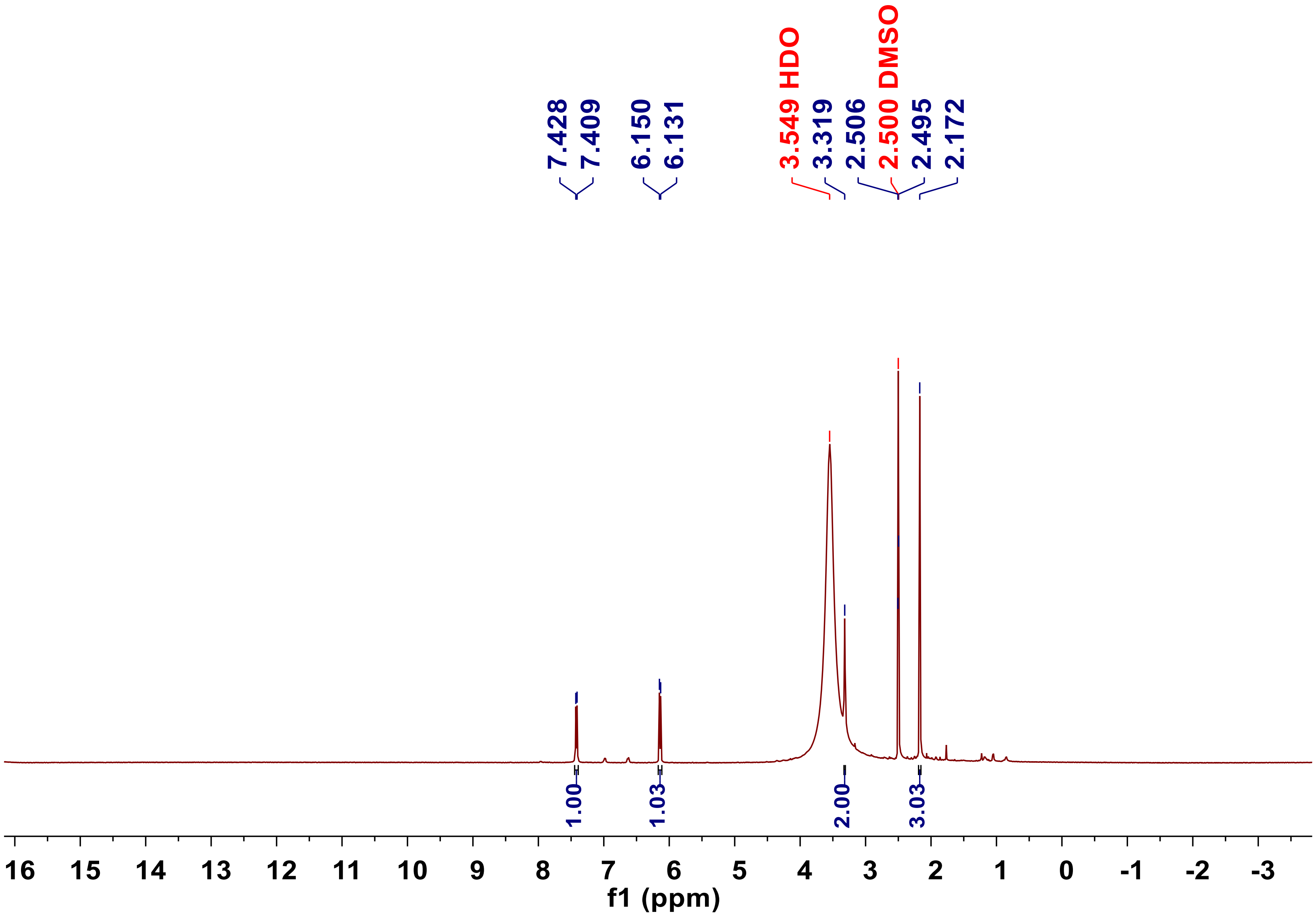

Figure S12 ^{13}C NMR spectrum of **4** in DMSO-d_6 (150 MHz)

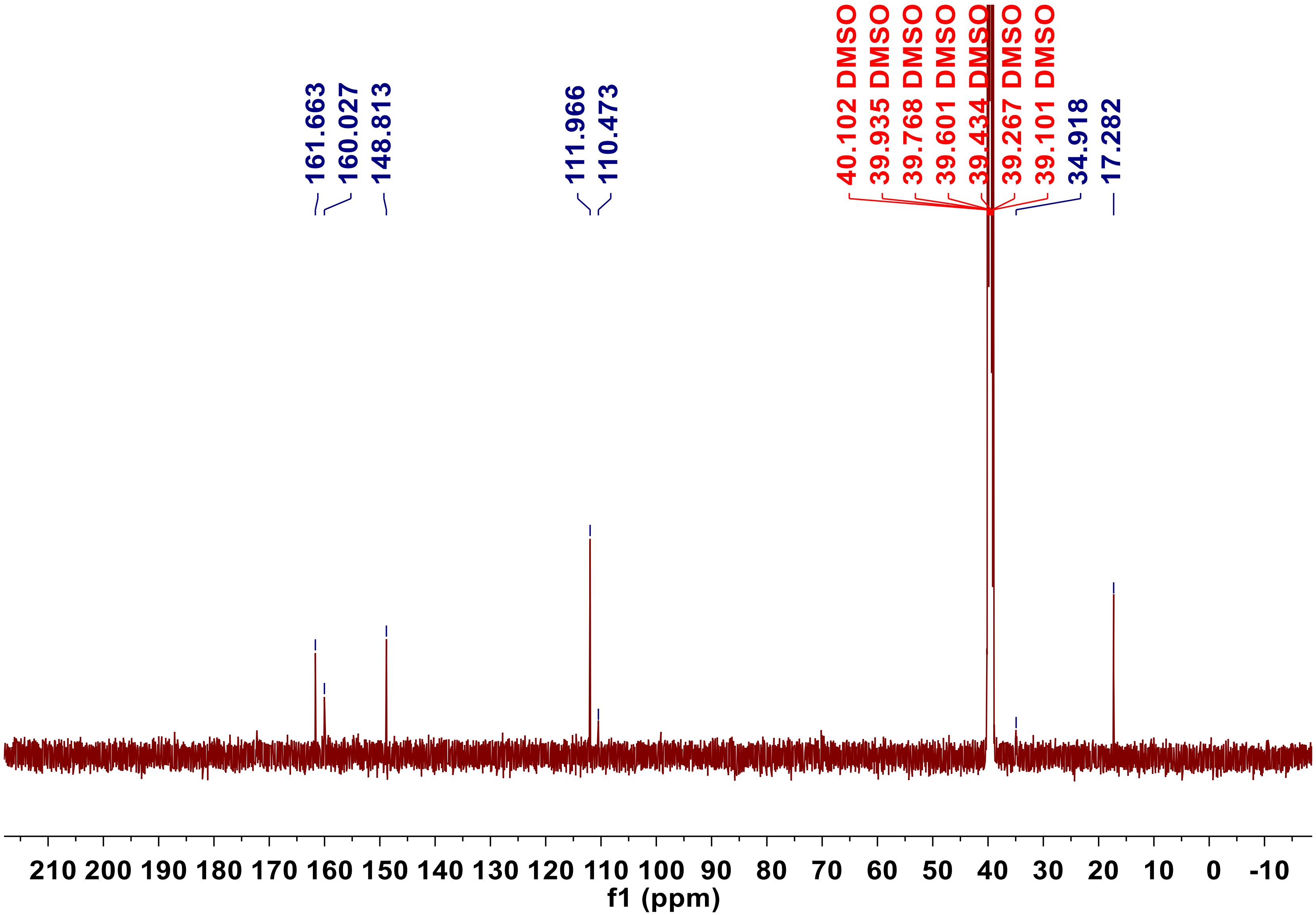

Figure S13 DEPT-135 spectrum of **4**

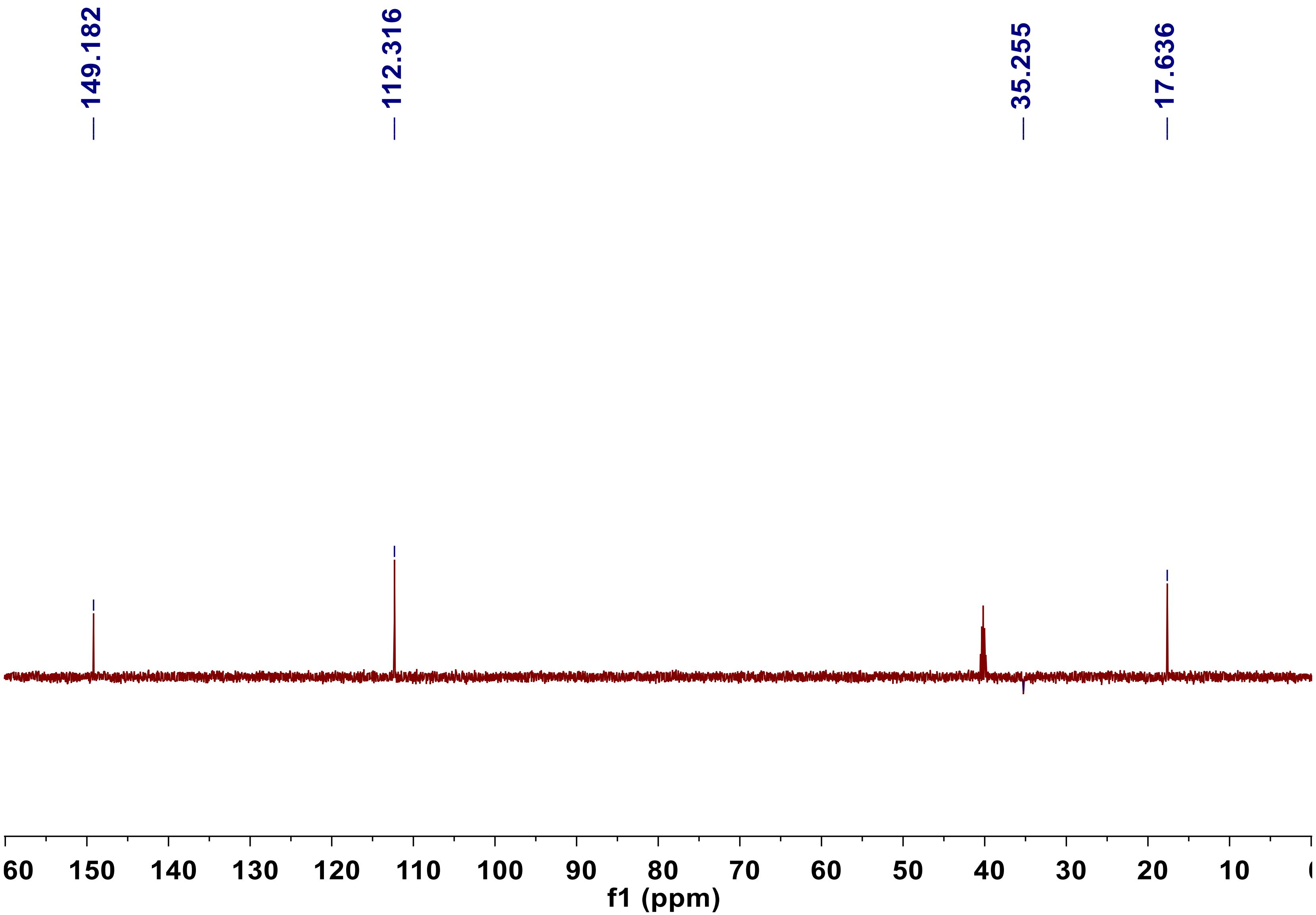

Figure S14 HSQC spectrum of **4**

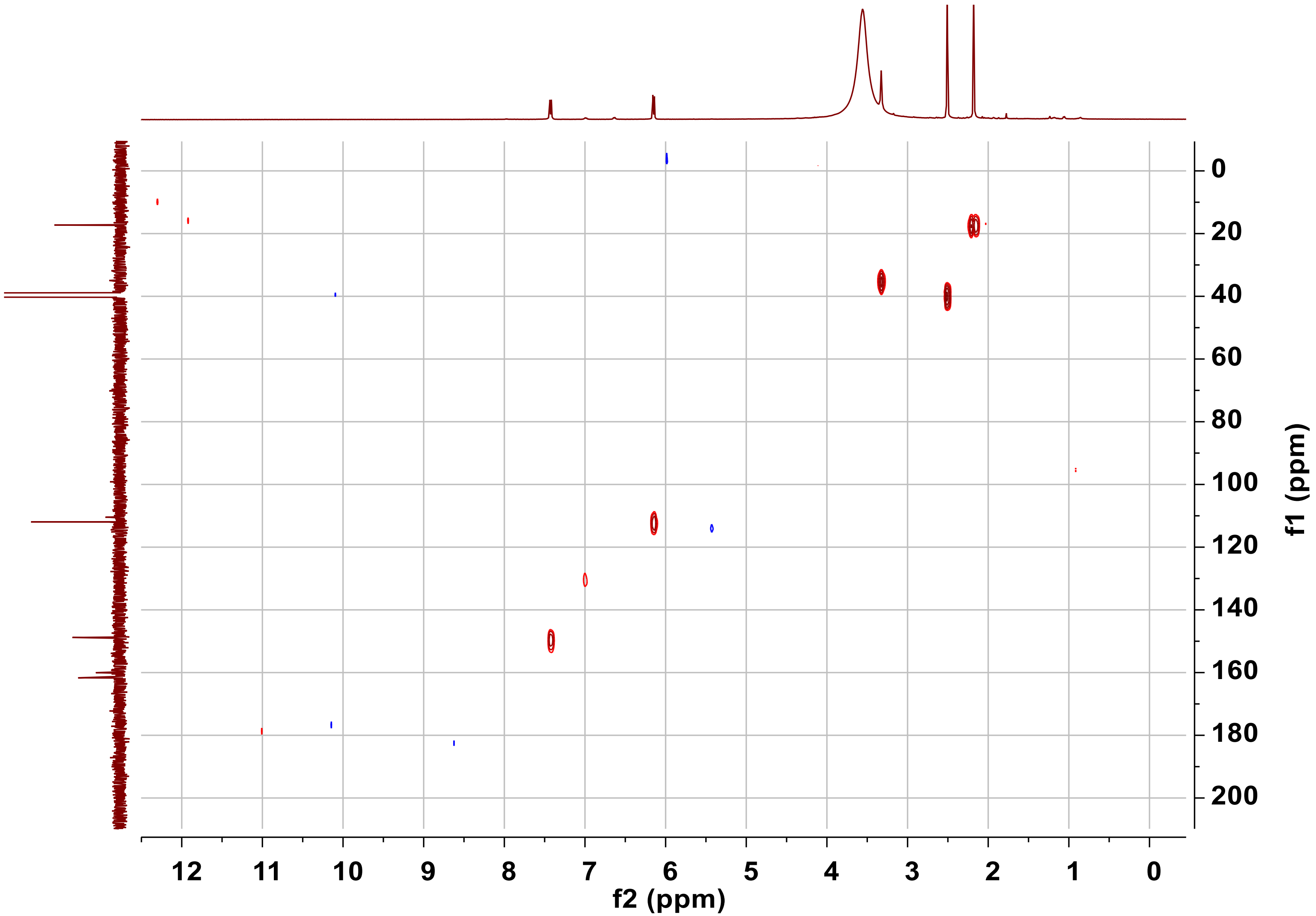

Figure S15 HMBC spectrum of **4**

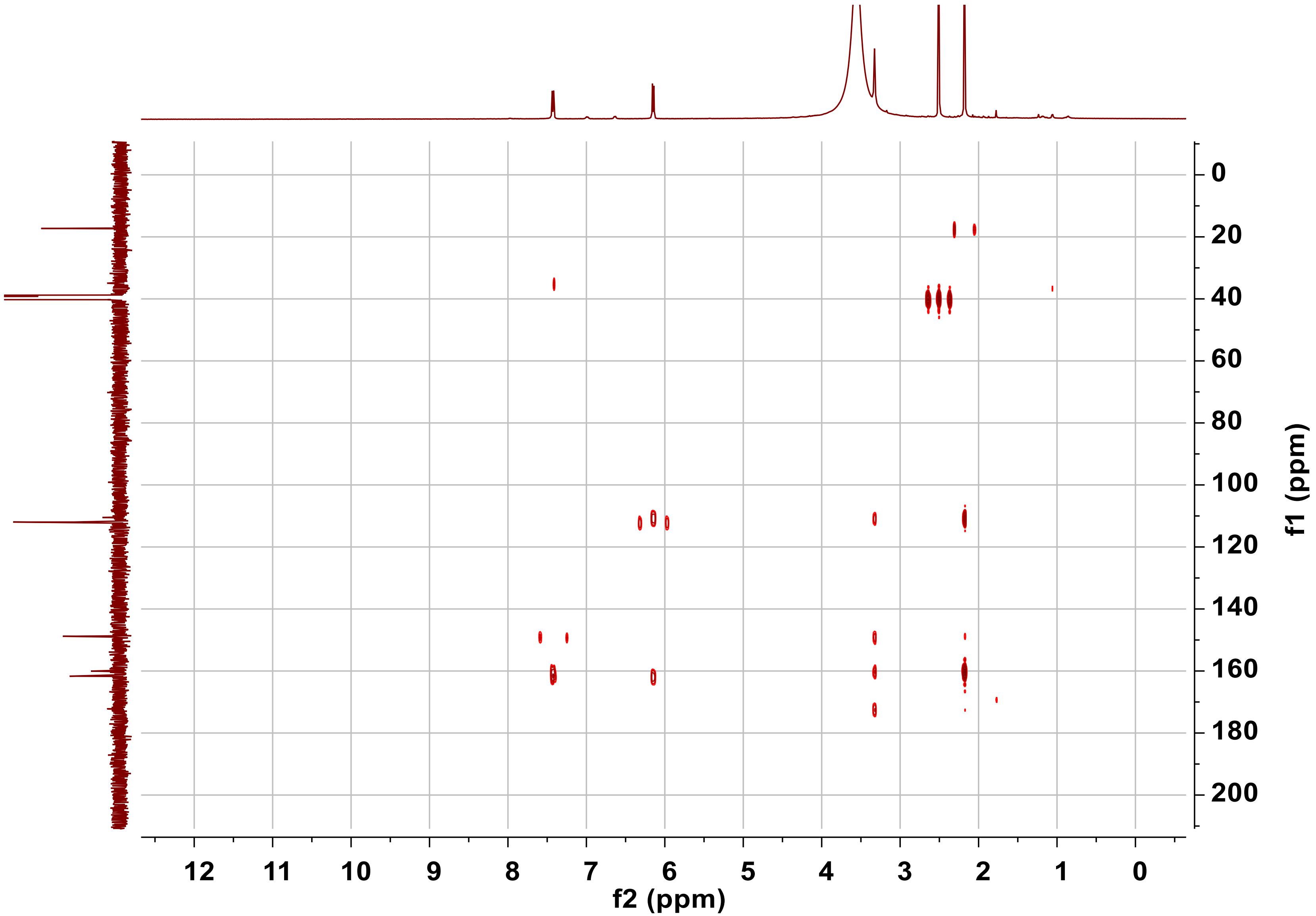

Figure S16 ^{1}H-^{1}H COSY spectrum of **4**

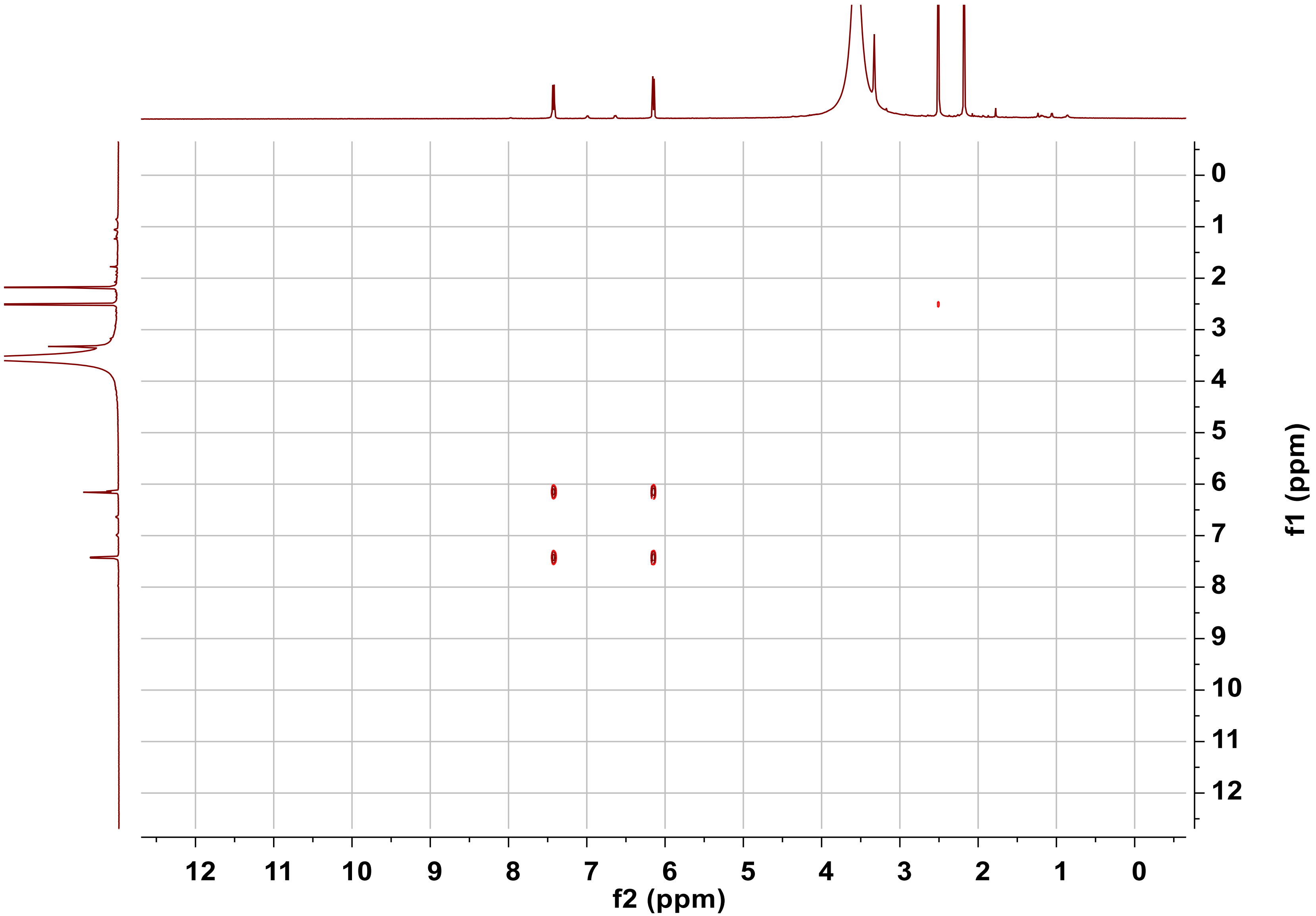

Figure S17 HRESIMS spectrum of **4**

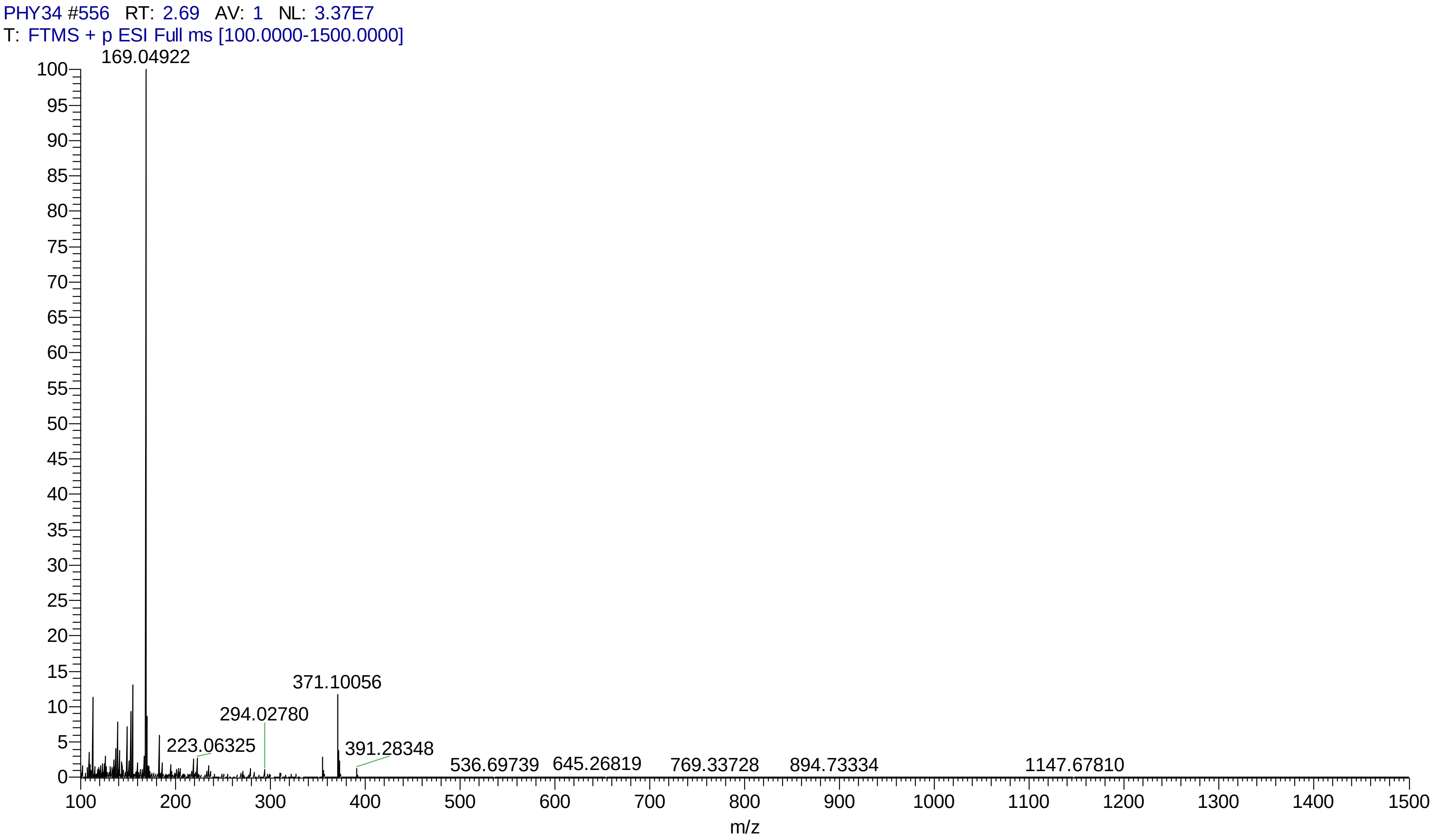

Figure S18 UV spectrum of **4**

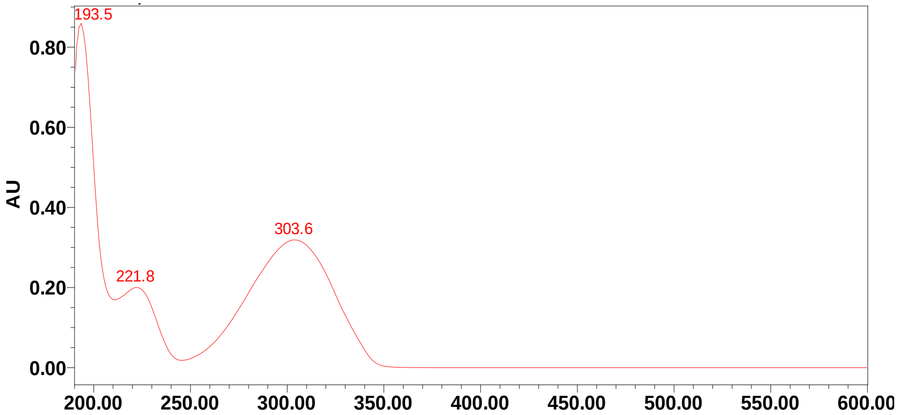

No.	Wavelength (nm)	Abs
1	193.5	0.860
2	221.8	0.200
3	303.6	0.319

Figure S19 Structures of diaporpyrones A-D (**S1-S4**) isolated from endophilic fungus strain *Diaporthe* sp. CB10100.

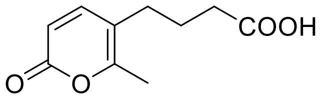

Diaporpyrone A (**S1**)

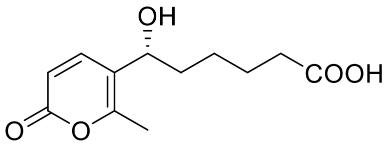

Diaporpyrone B (**S2**)

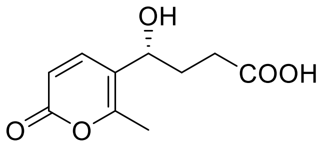

Diaporpyrone C (**S3**)

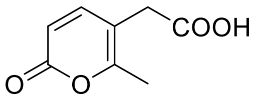

Diaporpyrone D (**S4**)

Figure S20 Docking poses and interactions of acarbose with α-glucosidase (PDB ID: 2QMJ).

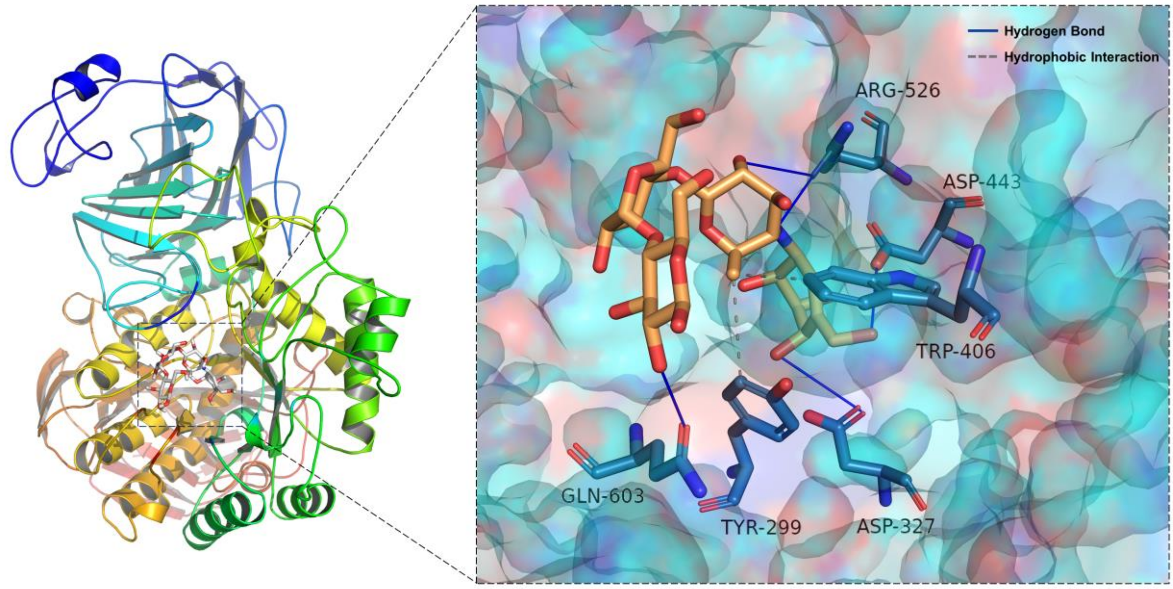

Figure S21 96 well plate assay of **3**-**4** against MRSA (**A**), *Mycolicibacterium (Mycobacterium) smegmatis* (**B**) and *Klebsiella pneumonia* (**C**) using the microbroth dilution method.

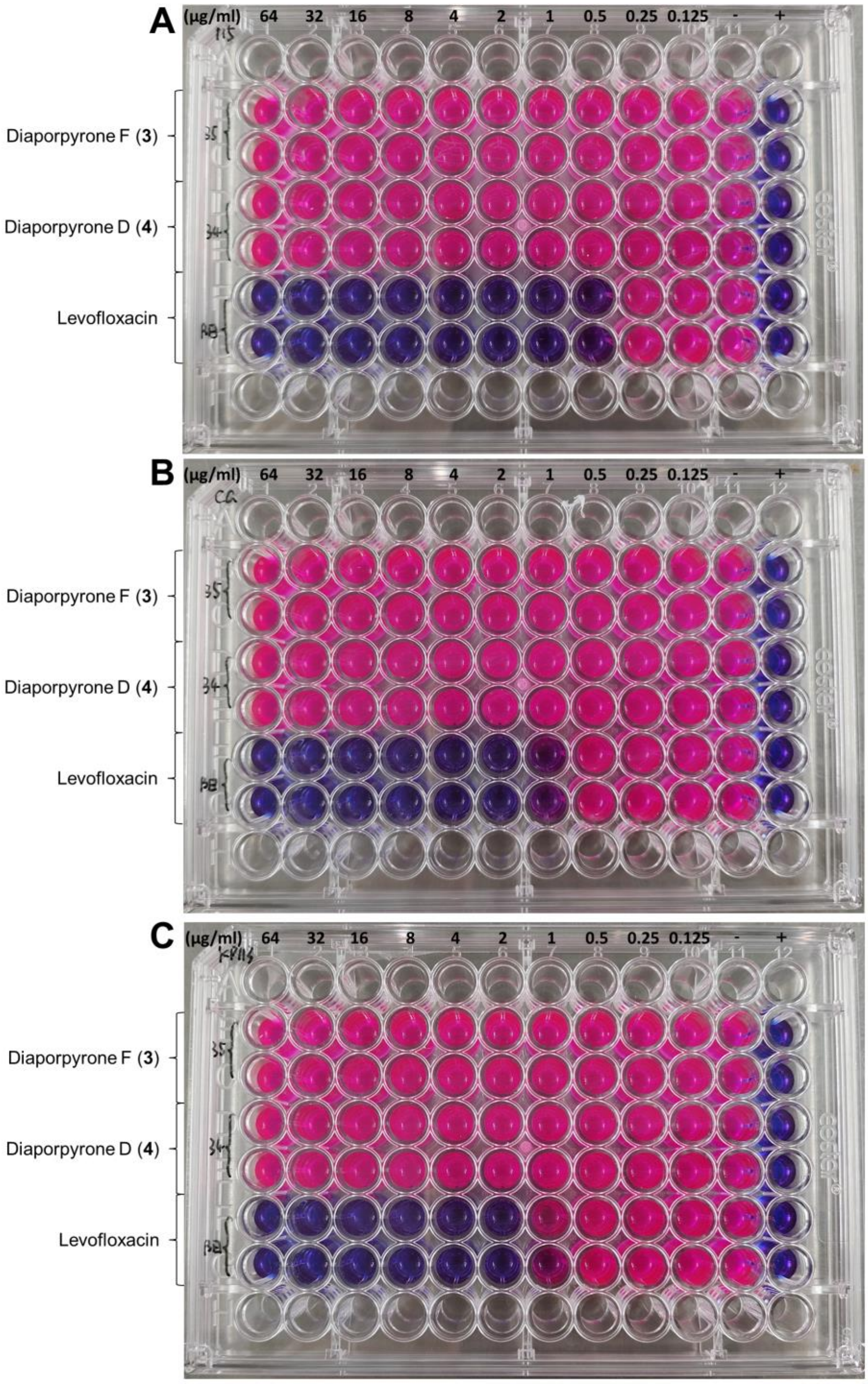

References

1. Xu, X.T.; Deng, X.Y.; Chen, J.; Liang, Q.M.; Zhang, K.; Li, D.L.; Wu, P.P.; Zheng, X.; Zhou, R.P.; Jiang, Z.Y.; et al. Synthesis and Biological Evaluation of Coumarin Derivatives as α-Glucosidase Inhibitors. *Eur. J. Med. Chem.* **2020**, *189*, 112013, doi:10.1016/j.ejmech.2019.112013.
2. Trott, O.; Olson, A.J. AutoDock Vina: Improving the Speed and Accuracy of Docking with a New Scoring Function, Efficient Optimization, and Multithreading. *J. Comput. Chem.* **2010**, *31*, 455–461, doi:10.1002/jcc.21334.
3. Sanner, M.F. Python: A Programming Language for Software Integration and Development. *J. Mol. Graph. Model.* **1999**, *17*, 57–61, doi: 10.1016/S1093-3263(99)99999-0.
4. Morris, G.M.; Huey, R.; Lindstrom, W.; Sanner, M.F.; Belew, R.K.; Goodsell, D.S.; Olson, A.J. AutoDock4 and AutoDockTools4: Automated Docking with Selective Receptor Flexibility. *J. Comput. Chem.* **2009**, *30*, 2785–2791, doi:10.1002/jcc.21256.
5. Salomon-Ferrer, R.; Case, D.A.; Walker, R.C. An Overview of the Amber Biomolecular Simulation Package. *WIREs Comput. Mol. Sci.* **2013**, *3*, 198–210, doi:10.1002/wcms.1121.
6. Sagui, C.; Darden, T.A. Molecular Dynamics Simulations of Biomolecules: Long-Range Electrostatic Effects. *Annu. Rev. Biophys. Biomol. Struct.* **1999**, *28*, 155–179, doi:10.1146/annurev.biophys.28.1.155.
7. Kräutler, V.; Van Gunsteren, W.F.; Hünenberger, P.H. A Fast SHAKE Algorithm to Solve Distance Constraint Equations for Small Molecules in Molecular Dynamics Simulations. *J. Comput. Chem.* **2001**, *22*, 501–508, doi:10.1002/1096-987X(20010415)22:5<501::AID-JCC1021>3.0.CO;2-V.
8. Larini, L.; Mannella, R.; Leporini, D. Langevin Stabilization of Molecular-Dynamics Simulations of Polymers by Means of Quasisymplectic Algorithms. *J. Chem. Phys.* **2007**, *126*, doi:10.1063/1.2464095.
9. Hou, T.; Wang, J.; Li, Y.; Wang, W. Assessing the Performance of the MM/PBSA and MM/GBSA Methods. 1. The Accuracy of Binding Free Energy Calculations Based on Molecular Dynamics Simulations. *J. Chem. Inf. Model.* **2011**, *51*, 69–82, doi:10.1021/ci100275a.
10. Chen, Y.; Zheng, Y.; Fong, P.; Mao, S.; Wang, Q. The Application of the MM/GBSA Method in the Binding Pose Prediction of FGFR Inhibitors. *Phys. Chem. Chem. Phys.* **2020**, *22*, 9656–9663, doi:10.1039/D0CP00831A.
11. Genheden, S.; Ryde, U. The MM/PBSA and MM/GBSA Methods to Estimate Ligand-Binding Affinities. *Expert Opin. Drug Discov.* **2015**, *10*, 449–461, doi:10.1517/17460441.2015.1032936.
12. Rastelli, G.; Rio, A.D.; Degliesposti, G.; Sgobba, M. Fast and Accurate Predictions of Binding Free Energies Using MM-PBSA and MM-GBSA. *J. Comput. Chem.* **2010**, *31*, 797–810, doi:10.1002/jcc.21372.
13. Nguyen, H.; Roe, D.R.; Simmerling, C. Improved Generalized Born Solvent Model Parameters for Protein Simulations. *J. Chem. Theory Comput.* **2013**, *9*, 2020–2034, doi:10.1021/ct3010485.
14. Weiser, J.; Shenkin, P.S.; Still, W.C. Approximate Atomic Surfaces from Linear Combinations of Pairwise Overlaps (LCPO). *J. Comput. Chem.* **1999**, *20*, 217–230, doi:10.1002/(SICI)1096-987X(19990130)20:2<217::AID-JCC4>3.0.CO;2-A.
